# A Scalable Similarity-Popularity Link Prediction Method

**DOI:** 10.1038/s41598-020-62636-1

**Published:** 2020-04-14

**Authors:** Said Kerrache, Ruwayda Alharbi, Hafida Benhidour

**Affiliations:** King Saud University, College of Computer and Information Sciences, Riyadh, 11543 Saudi Arabia

**Keywords:** Complex networks, Computational science, Computer science

## Abstract

Link prediction is the task of computing the likelihood that a link exists between two given nodes in a network. With countless applications in different areas of science and engineering, link prediction has received the attention of many researchers working in various disciplines. Considerable research efforts have been invested into the development of increasingly accurate prediction methods. Most of the proposed algorithms, however, have limited use in practice because of their high computational requirements. The aim of this work is to develop a scalable link prediction algorithm that offers a higher overall predictive power than existing methods. The proposed solution falls into the class of global, parameter-free similarity-popularity-based methods, and in it, we assume that network topology is governed by three factors: popularity of the nodes, their similarity and the attraction induced by local neighbourhood. In our approach, popularity and neighbourhood-caused attraction are computed directly from the network topology and factored out by introducing a specific weight map, which is then used to estimate the dissimilarity between non-adjacent nodes through shortest path distances. We show through extensive experimental testing that the proposed method produces highly accurate predictions at a fraction of the computational cost required by existing global methods and at a low additional cost compared to local methods. The scalability of the proposed algorithm is demonstrated on several large networks having hundreds of thousands of nodes.

## Introduction

The Internet, the World Wide Web, the brain and human society are some examples of systems that, despite seeming completely different at first sight, all share a fundamental property: they are all composed of interacting entities. Individual objects in these systems are not isolated, but rather connected through links or relationships. Mounting scientific evidence shows that these systems are better understood by investigating their properties as networks, where nodes represent individual components, and links refer to relationships, interactions or influences that exist among nodes^1^. Network science aims at understanding and creating effective tools for characterizing and quantifying complex systems. The first step in this endeavour is to observe and record the existing interactions in order to build the network. In most cases, however, it is not possible to observe all interactions between the individual components. This can be due either to limitations in the data collection process or because certain relationships have not yet been established^2^. The process of identifying the links that are missing from the network is known as *link prediction*. Recommending new friends or collaborators in social networks^3^, reconstructing networks^2^ and discovering unknown interactions in biological networks^4^ are few examples of the variety of applications that can benefit from predicting non-existing links.

Link prediction has proven to be a challenging problem, and a lot of efforts have been invested into the development of increasingly accurate prediction methods^5^. Often times, however, the improved accuracy comes at the price of a higher computational cost. The aim of this work is to develop a link prediction method that offers a high prediction quality at a reasonable computational requirement. The proposed method is inspired from the similarity-popularity model^6,7^. Our assumption is that the network topology is driven by the popularity of the nodes, their similarity as well as local attraction induced by their neighbourhood. In our approach, popularity and neighbourhood-caused attraction are deduced directly from the network topology, whereas similarity is estimated via shortest path distances. Extensive experimental evaluation shows that the proposed method produces highly accurate results at a computational cost that is several order of magnitude lower than that required by state-of-the-art global methods.

## Background and Related Work

The data for a link prediction problem consists simply in a network $$G(V,E)$$, where $$V$$ is the set of *nodes* and $$E$$ is the set of *edges*. We assume that the network $$G$$ contains no loops and that multiple edges between nodes are not allowed. We denote by $$U$$ the set of all possible links between the nodes of $$G$$. By definition, $$E\subseteq U$$, and if $$n$$ is the number of nodes in the graph (that is, $$n=| V| $$), then the set $$U$$ contains exactly $$n(n-1)/2$$ elements, which is the maximum number of undirected edges that can exist in the network. The set $$U-E$$ is the set of non-existent links, and the edges in this set are referred to as *negative edges*, whereas those in $$E$$ are called *positive edges*. The link prediction problem consists in discovering which elements of $$U-E$$ are missing from the network or may appear in the future^5^. This is typically achieved by assigning a score $${s}_{ij}$$ to every edge $$\left(i,j\right)$$ to be predicted. The higher score the more likely the edge actually exists.

Research on link prediction can be classified according to the type of information used in the prediction process into *topological-based* link prediction, where only the information available in the graph adjacency matrix is used, and *non-topological-based* (or *semantic*) link prediction, where the algorithms use node features in addition to the topological information to improve the prediction quality^8^. Link prediction methods can also be classified according to the type of model that is assumed to control the network topology. For instance, the model can be probabilistic^2^ or geometric^7^. Another important aspect that differentiates link prediction algorithms is the amount of topological information used to predict a single link. Global link prediction methods make use of the whole adjacency matrix to predict any given link, whereas local methods use only a small portion of the matrix to collect the local information necessary for prediction. Consequently, global methods tend to be computationally more intensive than their local counterparts.

### Topological ranking methods

The effectiveness of topological similarity measures in predicting links in networks was first extensively investigated in the work of Liben-Nowell and Kleinberg^9^. They proposed local and parameter-free approaches for link prediction, which have been later widely employed by the research community due to their simplicity, computational efficiency and performance. In their work, the authors compute likelihood scores for each pair of non-connected nodes using the common neighbours (CNE)^10^ index, Jaccard’s (JID)^11^ index, Adamic and Adar (ADA)^12^ index, hub promoted Index (HPI)^13^ and preferential attachment (PAT)^10^, and then rank the non-existing edges according to their scores, with the highest ranked edges being considered more likely to be connected. All these methods can be categorized as node-neighbourhood-based approaches except for PAT. By comparing the results obtained using different topological similarity indexes on five co-authorship networks, they concluded that topological information indeed improves link prediction performance. This work has motivated the introduction of several topological similarity measures for link prediction^14–17^. Zhou *et al*. introduced the resource allocation (RAL)^18^ index that, similarly to ADA index, gives more weight to nodes with low degrees. Zhu and Xia^19^ took the effort further by introducing an information-theoretic model that uses multiple topological features. The importance of each feature is determined by the value of information it contributes in deciding whether a link exits or not. Recently, inspired by a local-learning rule of neuron networks named Hebbian rule, Muscoloni and Cannistraci proposed Cannistraci-Hebb (CH)^20^ model (previously named Cannistraci-Ressourse-Allocation (CRA) rule^21,22^). The theory behind it is called *local community paradigm*, where two nodes are likely to be connected if their common neighbours are strongly connected, forming thus a local-community. CH model is a local, parameter-free deterministic model for link prediction in monopartite and bipartite networks that outperforms in general link-predictors that are considered as a reference.

### Semantic methods

Semantic methods combine topological information with content or semantic data related to nodes to improve prediction. In this setting, the link prediction problem is typically cast as a classification problem, where the classifier is trained to discriminate between positive instances (connected couples) and negative instances (disconnected couples). To predict missing links, Hasan *et al*.^3^ used topological features, such as the shortest path distances, in addition to several semantic features, for instance, the number of matching keywords. Their experimental results show that semantic similarity improves the accuracy of the link prediction. In addition to topological and semantic similarity, Wang *et al*.^23^ used the joint co-occurrence probability of nodes as a feature and used a logistic regression classifier to predict links based on these features. Their experiments show that the performance is improved when all three features are combined.

Although proven useful, semantic data may not always exist, may be imprecise, or in many cases difficult to collect. Another limitation of semantic methods is that they require domain-knowledge to select the features to be used.

### Probabilistic methods

Probabilistic models have been particularity successful in solving the link prediction problem. Among these models, global prediction frameworks based on the community structure of the network have been proposed by Clauset *et al*. in^4^, and by Guimerà and Sales-Pardo in^2^. Clauset *et al*. introduced a Hierarchical Random Graph model (HRG)^4^ that can be used to predict the existence of links between the graph nodes. The hierarchical structure consists of a binary tree with leaves representing the nodes of the network. Internal nodes, on the other hand, correspond to nested clusters, each associated with the probability of a link existing between any two of its children. Hence, the probability that two nodes are connected can be determined by locating their lowest common ancestor in the tree. This method was used successfully to predict links in partially known social and biological networks. In^2^, the authors introduced Stochastic Block Model (SBM) in which the nodes are partitioned into groups, and the probability of existence of a link between two nodes depends on the groups to which they belong. Their mathematical framework allows to capture the community structure and estimate links reliability. The latter describes the probability that a link exists, and in addition to predicting missing links, it can also be used to identify spurious ones. It is worth mentioning that due to combinatorial explosion, the use of these two global probabilistic methods (HRG and SBM) is in practice limited to small-sized networks^5^. Liu *et al*. presented Fast Blocking probabilistic Model (FBM)^24^, a greedy-based approach in which link densities within and among communities are used to estimate the probability of a link. HRG, SBM and FBM are global link predictors that use only the topological information (adjacency matrix) for the prediction. FBM has significantly less computational complexity comparing to HRG and SBM.

### Similarity-popularity models

Similarity-popularity models^6,7^ are a family of complex network models that ascribe the network topology to two properties: the similarity between nodes and their popularity. Boguna *et al*.^6^ proposed a hidden variables model that assumes the existence of a hidden metric space underlying real networks. This metric space encodes the similarity between nodes: nodes that are at a smaller distance from one another exhibit more similarity than those that are at a larger distance. Additionally, each node is assigned a hidden degree $\kappa$ that represents its popularity. These hidden degrees are drawn from a power-law degree distribution $P(k) \sim {k}^{-\gamma }$, with $\gamma  > 2$. The probability that a pair of nodes $i$ and $j$ are connected is then given by^6^:

1 $$r(d;{\kappa }_{i},{\kappa }_{j})={(1+d/{d}_{c})}^{-\alpha },$$

where $d$ is the distance between node $i$ and $j$, ${d}_{c} \sim {\kappa }_{i}{\kappa }_{j}$ with ${\kappa }_{i}$ and ${\kappa }_{j}$ being the expected degrees of $i$ and $j$, and $\alpha  > 1$.

A direct implication of Eq. (1) is that the connection probability is inversely proportional to the distance between the nodes. Hence, all other things being equal, similar nodes tend to connect more than dissimilar nodes. The connection probability is one the other proportional to the product of the hidden degrees of the two nodes: if all other things are kept equal, then more popular nodes have more chance to connect than less popular nodes. The connection probability is therefore a trade-off between the two properties, similarity and popularity. In^25^, the authors investigated a variation of the hidden metric space model in which the underlying space possesses a hyperbolic geometry, and in^26^, HyperMap (HYP), a method for estimating the parameters of the hyperbolic model from data is presented. The estimated model can then be used to compute the probability of existence of links between any pair of nodes in the network.

## Results

In this paper, we generalize the model proposed in^6^ and assume that in addition to similarity and popularity, the likelihood of existence of a link between two given nodes also depends on the attraction forces induced by their local neighbourhood. Namely, we assume that the likelihood of a link between two nodes $i$, $j$ is proportional to:

2 $$\Psi (i,j)=\left({\pi }_{ij}+{\eta }_{ij}\right){s}_{ij},$$

where ${s}_{ij}$ is the similarity between $i$ and $j$, ${\pi }_{ij}$ is a measure of the popularity of the two nodes, and ${\eta }_{ij}$ represents the attraction between the nodes caused by their local neighbourhood. The popularity term ${\pi }_{ij}$ reflects the tendency of the two nodes to connect to other nodes in the networks and is therefore chosen to be an increasing function of the nodes’ degrees. There are many possible choices for ${\pi }_{ij}$, but in this work we propose a formula that will prove to scale well for large networks. First, let the function $\phi$ be defined as:

3 $$\phi (x)={\rm{\log }}\,(x+1).$$

The popularity term ${\pi }_{ij}$ is then given by:

4 $${\pi }_{ij}=\frac{\phi ({\kappa }_{i})+\phi ({\kappa }_{j})}{2\phi ({\kappa }_{\max })},$$

where ${\kappa }_{i}$ and ${\kappa }_{j}$ are the degrees of $i$ and $j$ respectively, and ${\kappa }_{max}$ is the maximum degree in the network. The purpose of adding the constant one inside the logarithm in the definition of $\phi$ is to avoid indefiniteness when dealing with isolated nodes (having degree 0). The denominator in Eq. (2) is used for normalization, so that the value ${\pi }_{ij}$ lies in the interval $[0,1]$. Unlike the popularity term ${\pi }_{ij}$, which depends only on the degrees of the two nodes, ${\eta }_{ij}$ depends on the local topology in the vicinity of the two nodes:

5 $${\eta }_{ij}=1-\prod _{k\in {\Gamma }_{ij}}\frac{\phi ({\kappa }_{k})}{\phi ({\kappa }_{\max })},$$

where ${\Gamma }_{ij}$ is the set of common neighbours of $i$ and $j$, and ${\kappa }_{k}$ is the degree of node $k$. By convention ${\eta }_{ij}$ is set to 0 if ${\Gamma }_{ij}$ is empty. Note that, similarly to ${\pi }_{i,j}$, ${\eta }_{ij}\in [0,1]$ and is larger when $i$ and $j$ are connected via low degree nodes compared to when being connected by neighbours having high degrees. Finally, the similarity term ${s}_{ij}$ can be written as a function of the hidden distance between the two nodes as follows:

6 $${s}_{ij}=\frac{1}{1+{d}_{ij}}.$$

Hence, ${s}_{ij}\in [0,1]$, with ${s}_{ij}=0$ for nodes at infinite distance, and it is equal to 1 for nodes at zero distance. Except for the similarity term ${s}_{ij}$, all quantities in Eq. (2) can be easily computed given the observed network. The distance, or dissimilarity, on the other hand, must be inferred from the network topology. To achieve this, we proceed as follows: for every edge $\left(i,j\right)\in E$, we assign the length $\omega (i,j)$ given by:

7 $$\omega (i,j)=\frac{2{\pi }_{ij}}{1+{\eta }_{ij}},$$

**Figure 1:**
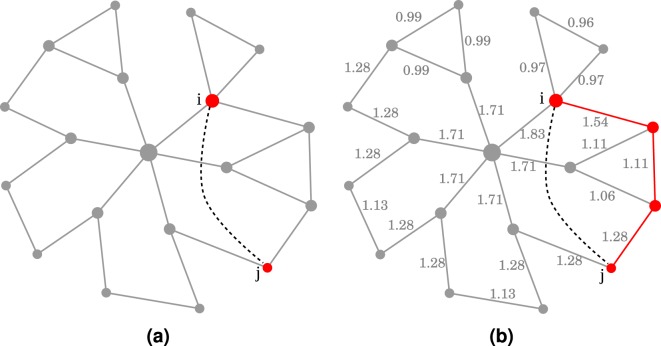
Example of link prediction using Algorithm 1. (**a**) Shows the input network. The algorithm assigns to each disconnected couple a score representing the likelihood of the existence of an edge between these nodes. To compute the score ${\psi }_{ij}$ for the disconnected couple $(i,j)$, the network is first weighted using Eq. (7) as shown in (**b**). The distance ${d}_{ij}$ is then estimated as the length of the shortest path between $i$ and $j$ (highlighted in red). Notice that the weight map causes the shortest path to pass through the peripheral small-degree nodes instead of the central hub node. Finally, the score is assigned to $(i,j)$ according to Eq. (2).

In this formula, the length of an edge is inversely related to local attraction. This is consistent with the phenomenon of clustering caused by hidden metric spaces: nodes with strong local attraction forces are more likely to be at a short distance in the hidden space compared to nodes with weak local attraction. The nominator in Eq. (7) has for role to factor out the popularity effect: popular nodes do not require a short distance to connect and can therefore be assigned a large edge length. Unpopular nodes, on the other hand, are assigned low edge length to enable them to connect. Using the obtained weight map, it is possible to approximate the dissimilarity between non-adjacent nodes by their shortest path distance. Once computed, the latter can be used to assign a score ${\psi }_{ij}=\Psi \left(i,j\right)$ to any negative link $(i,j)$. The higher the score ${\psi }_{ij}$ the more likely that a link exists between $i$ and $j$. The proposed link prediction procedure is therefore a global and parameter-free similarity-popularity-based predictor. Figure 1 shows an example of computing the score ${\psi }_{ij}$ for a disconnected couple of nodes $(i,j)$ using the proposed approach.

From the computational perspective, the critical step in the proposed method is the computation of the shortest path distances. If the set of links to be predicted is small, it is possible to use Dijkstra’s algorithm. If, on the other hand, the prediction involves all disconnected couples in the network, we end up with the all-couples shortest path problems, which can be solved in $O({n}^{3})$ using Floyd-Warshall’s algorithm. Since most real networks are sparse, however, it is in general more efficient to run Dijkstra’s algorithm $n$ times, which results in an overall running time of $O(nm+{n}^{2}{\rm{\log }}\,m)$, where $m$ is the number of edges. This is computationally better than running Floyd-Warshall’s algorithm if the network is sparse. A more efficient approach for reducing the running time is to impose a horizon cut-off when running Dijkstra’s algorithm. In this setting, given a horizon cut-off $h$, all paths having more than $h$ edges are discarded. Consequently all nodes that cannot be connected via paths having at most $h$ edges are considered disconnected and are assigned an infinite distance. The usual definition of shortest path distances corresponds to $h=\infty$. Using a small value for $h$ can dramatically reduce the computation time and, as we shall see in the experimental evaluation, causes virtually no reduction in performance in real networks. This renders the method scalable for very large networks as demonstrated by the experimental results. The proposed method is summarized in Algorithm 1. There, $ShortestPathDistance(V,E,\{{w}_{ij}\},h)$ computes the shortest path distances between nodes in the graph $(V,E)$ using ${w}_{ij}$ as weights and $h$ as horizon cut-off.

**Algorithm 1:**
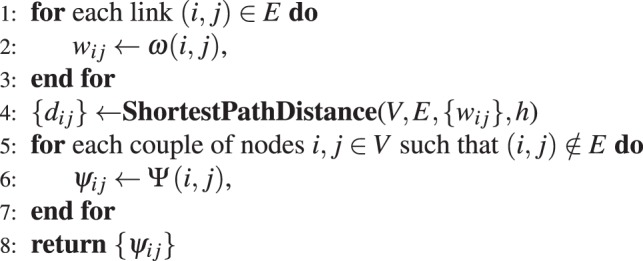
.

### Experimental evaluation

To assess the performance of the proposed algorithm (referred henceforth as ALG1), we conduct a series of experiments on synthesized and real network data. The first experiment consists in assessing the effect of the horizon cut-off on the predictive power of Algorithm 1. We then compare the proposed method to a set of well known global link prediction methods that have high accuracy in general but are in most cases computationally intensive, and hence are not scalable to large networks. Comparison to local methods, which are fast and scalable in general, is conducted next on simulated and real networks of different types and sizes. We also investigate the effect of network topological properties on the proposed algorithm. The last experiment compares the time performance of the proposed algorithm to existing methods and demonstrates its scalability. In the next sections, the performance metrics used for the evaluation are presented followed by a description of the experiments conducted.

#### Performance metrics

The test data used to evaluate the performance of link prediction algorithms is created from ground truth networks by removing a set of links that is then used as a test set. As customary in link prediction literature, we shall use a fixed and small removal rate of 10%. In other words, the prediction algorithm is presented with 90% of the links, whereas the remaining 10% are used to assess its predictive power. Traditionally, the main performance measure used to evaluate the performance of link prediction methods has been the area under the receiver operating curve (AUROC)^5^, which can be computed as the probability that a false negative link (that is, a removed link) is assigned a score that is higher than that of a true negative link (a negative link in the original network). However, despite the fact that the AUROC metric is unbiased for imbalanced datasets, recent studies^18,20,27,28^ have pointed out that it is unsuitable to use it for evaluating link prediction algorithms. Link prediction problems are characterized by a large skew within the class distribution, particularly in sparse networks. The size of the negative set, defined as the set of non-existent edges except the removed ones used for the test, is much larger than the set of the positive set, defined as the edges removed for the test. Using the area under ROC curve, especially in large networks, can provide an overly-optimistic view of the performance, since the rate of mistakes comparing to the negative set size can hide their actual magnitude^27,29^. The area under the precision-recall curve (AUPR) metric can provide a better evaluation when dealing with imbalanced datasets^29^, since it considers only the performance of the positive set. This is particularly true for link prediction^18^, where the positive set is usually very small compared to the negative set. Another performance measure that avoids the pitfalls of the AUROC is top-precision (TPR), also referred to as top-$k$ predictive rate or r-precision^18^, and sometimes simply (although inaccurately) as precision. This measure has been adopted as a performance metric in recent works on link prediction^20,28^. After ranking the non-existent links according to the prediction score, top-precision is computed as the percentage of positive links (the ones removed to be used as the test set) within the top $k$ ranked links, where $k$ is usually taken as the total number of removed links^5,18^.

In this paper, we adopt top-precision as the main metric for measuring the performance of link prediction algorithms. As we are using multiple datasets to compare the performance of the algorithms, we also need to aggregate results obtained using different networks. Since these networks exhibit very different topological properties, simply averaging the results may lead to erroneous conclusions. To remedy this problem, we will use an adapted version of the top-precision-ranking metric proposed recently in^20,28^. Instead of simply ranking the algorithms as originally proposed in^20,28^, we conduct a statistical test for the results of each network and only consider statistically significant differences. More precisely, for each network, we conduct a two-tailed paired t-test for each couple of algorithms. If the results of the two algorithms are not statistically significantly different at the specified confidence level, both algorithms are assigned the score 0 for this network. If, on the other hand, the results are statistically significantly different, the algorithm with the better results is assigned the score 1, whereas the other algorithm is assigned -1. These pairwise scores are summed to obtain a network score for every algorithm. The algorithms are then decreasingly ranked based on these network scores and in case of a tie, the average rank is used. The final evaluation score of an algorithm is computed as the average rank over all networks. In what follows, we shall refer to this average as the *average significant rank*.

#### The effect of the horizon cut-off

**Figure 2:**
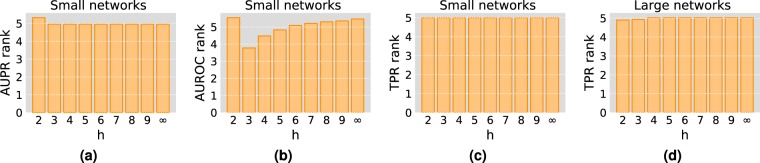
The effect of the horizon cut-off $h$ on the performance of Algorithm 1. We report the average significant ranks at $p=0.05$ using different values of $h$. In (**a–c**), we show the ranks based on the area under the PR curve (AUPR), the area under the ROC curve (AUROC) and top-precision (TPR) respectively. These results are obtained using 40 small networks. The average top-precision significant ranks on a set of 40 large networks are shown in (**d**).

To assess the effect of the horizon cut-off $h$ on the performance of the proposed method, we run the algorithm with different values of $h$ on several real networks and compare the results. For each network, we randomly remove 10% of the edges and set them aside as test set. In this experiment, we use two sets of networks: one consisting of 40 small networks (having less than 1000 nodes), and the other containing 40 large networks (having more than 1000 nodes). The number of test runs per network is 1000 for small networks and 100 for large networks. For small networks, we compute the average significant ranks at $p=0.05$ based on AUPR, AUROC and top-precision. For large networks, we limit the results to top-precision as it is our main performance measure and also to keep the computation time reasonable. Figure 2 shows the obtained average significant ranks (detailed per-networks results can be found in Tables 4–7 of SI). As can be seen in Fig. 2(c,d), top-precision is essentially insensitive to the variations of $h$. The same applies to a large extent to AUPR (Fig. 2(a)), except for $h=2$ which seems to be the only value that gives lower quality results. The AUROC, on the other hand, is sensitive to changes in the horizon cut-off as can be seen from Fig. 2(b), where we can see that $h=3$ produces the best results. Since we are mainly concerned with top precision, however, we shall use the value $h=2$ in the remaining experiments as it produces the same quality as higher values of $h$ but at a much lower computational cost as it is demonstrated in the time performance experiment at the end of this section.

#### Comparison against global methods

**Figure 3:**
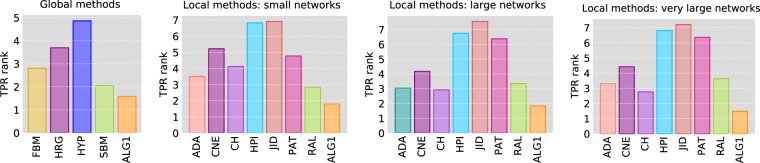
Comparison of Algorithm 1 against global and local link prediction methods on real networks. We show the average significant rank at $p=0.05$ based on top-precision (the lower the better). Comparison against global methods is done using 18 small networks. For local methods, we use 40 small networks (with up to 1000 nodes), 40 large networks (with up to 30,000 nodes) and 26 very large networks (with up to 400,000 nodes).

In this experiment, we compare the performance of the proposed approach against a set of well-known global link prediction algorithms, namely: Hierarchical Random Graph (HRG)^4^, Stochastic Block Model (SBM)^2^, Fast Block probabilistic Model (FBM)^24^ and HyperMap (HYP)^26^ (see Background and Related Work section for a description of these algorithms). All these methods use only the topological information (adjacency matrix) for predicting the links. They are also known for producing fairly accurate predictions, but this often comes at a high computational cost. The comparison is conducted on 18 small real networks of various types and orders ranging from 35 nodes (DNA Citation network) to 643 nodes (Political Blog network). The size restriction is essentially due to the high computational cost of the global methods used for comparison. The average significant ranks obtained with a $p$-value of 0.05 are shown in Fig. 3 (left), whereas the detailed per-network results are reported in Table 8 of SI. The results show that the proposed method performs consistently better against the other methods. It has the best average significant rank although the statistical significance tests reported in Table 1 show that the superiority against SBM is not statistically significant at 95% confidence level. As shown in the time performance experiment, this level of performance of the proposed method is achieved at a computational cost that is a mere fraction of what the other global methods require including SBM. Indeed, most real-life networks are large in size, which makes the availability of scalable link prediction methods such such as the proposed algorithm crucial for practical applications.

**Table 1:** Results of statistical significance test of the difference in mean significant ranks. The results reported are those of the two-sample, one-tailed Mann-Whitney-Wilcoxon test of the mean significant ranks based on top-precision. For each entry, the upper triangle of the table reports the test p-values adjusted using the Benjamini, Hochberg, and Yekutieli method. Results significant at 95% confidence level are reported in boldface text. The lower triangular part reports, when significant at 95% confidence level, the comparison of the average significant rank with $p=0.05$ between the algorithm in the row and the one in the column.

Global methods																	
	FBM	HRG	HYP	SBM	ALG1												
FBM		**0.01**	**0.00**	0.08	**0.00**												
HRG	>		**0.00**	**0.00**	**0.00**												
HYP	>	>		**0.00**	**0.00**												
SBM		<	<		0.21												
ALG1	<	<	<														
**Local methods on real networks**									**Local methods on simulated networks**								
**Small networks**									**nPSO networks**								
	ADA	CNE	CH	HPI	JID	PAT	RAL	ALG1		ADA	CNE	CH	HPI	JID	PAT	RAL	ALG1
ADA		**0.00**	0.26	**0.00**	**0.00**	0.09	**0.05**	**0.00**	ADA		**0.00**	0.29	**0.00**	**0.00**	**0.00**	**0.00**	**0.00**
CNE	>		**0.00**	**0.00**	**0.00**	1.00	**0.00**	**0.00**	CNE	>		**0.00**	**0.00**	**0.00**	**0.00**	**0.00**	**0.00**
CH		<		**0.00**	**0.00**	0.40	**0.00**	**0.00**	CH		<		**0.00**	**0.00**	**0.00**	**0.00**	**0.00**
HPI	>	>	>		0.59	**0.00**	**0.00**	**0.00**	HPI	>	>	>		**0.00**	**0.00**	**0.00**	**0.00**
JID	>	>	>			**0.00**	**0.00**	**0.00**	JID	>	>	>	>		**0.00**	**0.00**	**0.00**
PAT				<	<		**0.02**	**0.00**	PAT	>	>	>	>	>		**0.00**	**0.00**
RAL	<	<	<	<	<	<		**0.00**	RAL	<	<	<	<	<	<		0.42
ALG1	<	<	<	<	<	<	<		ALG1	<	<	<	<	<	<		
**Large networks**									**Watts-Strogatz networks**								
	ADA	CNE	CH	HPI	JID	PAT	RAL	ALG1		ADA	CNE	CH	HPI	JID	PAT	RAL	ALG1
ADA		**0.00**	1.00	**0.00**	**0.00**	**0.00**	0.93	**0.00**	ADA		**0.00**	**0.00**	**0.00**	**0.00**	**0.00**	0.08	**0.00**
CNE	>		**0.00**	**0.00**	**0.00**	**0.00**	**0.05**	**0.00**	CNE	<		**0.00**	**0.00**	**0.00**	**0.00**	**0.00**	**0.00**
CH		<		**0.00**	**0.00**	**0.00**	0.67	**0.00**	CH	>	>		**0.00**	**0.00**	**0.00**	**0.00**	**0.00**
HPI	>	>	>		**0.00**	0.30	**0.00**	**0.00**	HPI	<	<	<		0.30	**0.00**	**0.00**	**0.00**
JID	>	>	>	>		**0.00**	**0.00**	**0.00**	JID	<	<	<			**0.00**	**0.00**	**0.00**
PAT	>	>	>		<		**0.00**	**0.00**	PAT	>	>	>	>	>		**0.00**	**0.00**
RAL		<		<	<	<		**0.00**	RAL		>	<	>	>	<		**0.00**
ALG1	<	<	<	<	<	<	<		ALG1	>	>	>	>	>	<	>	
**Very large networks**									**Barabàsi-Albert networks**								
	ADA	CNE	CH	HPI	JID	PAT	RAL	ALG1		ADA	CNE	CH	HPI	JID	PAT	RAL	ALG1
ADA		**0.00**	0.05	**0.00**	**0.00**	**0.00**	1.00	**0.00**	ADA		**0.00**	**0.00**	**0.00**	**0.00**	**0.00**	**0.00**	**0.01**
CNE	>		**0.00**	**0.00**	**0.00**	**0.00**	0.30	**0.00**	CNE	<		**0.00**	**0.00**	**0.00**	**0.00**	**0.00**	0.53
CH		<		**0.00**	**0.00**	**0.00**	0.15	**0.00**	CH	>	>		**0.00**	**0.00**	**0.00**	1.00	**0.00**
HPI	>	>	>		0.18	1.00	**0.00**	**0.00**	HPI	>	>	>		**0.00**	**0.00**	**0.00**	**0.00**
JID	>	>	>			0.37	**0.00**	**0.00**	JID	>	>	>	>		**0.00**	**0.00**	**0.00**
PAT	>	>	>				**0.00**	**0.00**	PAT	<	<	<	<	<		**0.00**	**0.00**
RAL				<	<	<		**0.00**	RAL	>	>		<	<	>		**0.00**
ALG1	<	<	<	<	<	<	<		ALG1	<		<	<	<	>	<	
**CAIDA AS networks**																	
	ADA	CNE	CH	HPI	JID	PAT	RAL	ALG1									
ADA		**0.00**	**0.00**	**0.00**	**0.00**	**0.00**	**0.00**	**0.00**									
CNE	>		**0.00**	**0.00**	**0.00**	**0.00**	**0.00**	**0.00**									
CH	<	<		**0.00**	**0.00**	**0.00**	**0.00**	**0.00**									
HPI	>	>	>		**0.00**	**0.00**	**0.00**	**0.00**									
JID	>	>	>	>		**0.00**	**0.00**	**0.00**									
PAT	>	>	>	<	<		**0.00**	**0.00**									
RAL	>	>	>	<	<	<		**0.00**									
ALG1	<	<	<	<	<	<	<										

#### Comparison against local methods on real networks

We evaluate the performance of the proposed method by comparing it to 12 local prediction algorithms. In this section and subsequent ones, we report the results of a representative sample of these methods consisting of: Adamic-Adar index (ADA), common neighbours (CNE), Cannistraci-Hebb model (CH), hub promoted index (HPI), Jaccard index (JID), preferential attachment (PAT) index, and resource allocation index (RAL). All the methods used for comparison use only the local graph information to rank the disconnected couples and therefore have a running time that is $O({n}^{2})$ making them easily scalable to large networks^5,30^. More than a hundred networks of various types and with sizes ranging from less than 50 nodes to several hundred thousands nodes are used in the evaluation process. Figure 3 shows the average significant ranks based top-precision obtained using three sets of networks: a set of 40 small networks having less than 1000 nodes, a set of large networks having more than 1,000 nodes and less than 30,000 nodes, and one consisting of 26 very large networks having up to 400,000 nodes. Table 2 shows the average top-precision results for a sample of 12 networks. The full detailed results are reported in Tables 9–12 of SI. As the results show, the proposed method gives the highest top-precision in the majority of the networks and has the lowest average significant rank in all three types of networks. This is further confirmed by the results of the statistical significance tests reported in Table 1.

**Table 2:** Sample results of comparison between Algorithm 1 and local link prediction methods on real networks (full results in SI). We report top-precision obtained using 1000 test runs in the case of small networks and 100 test runs for larger networks. In each test run 10% of the edges are removed and used as test set. For every network, the results having the best significant rank with $p=0.05$ are shown in bold. The columns $n$ and $m$ show the number of nodes and edges of the networks.

Network	$n$	$m$	ADA	CNE	CH	HPI	JID	PAT	RAL	ALG1
Terrorist	62	152	**0.2923**	0.2443	0.2762	0.1568	0.0549	0.1059	**0.2907**	**0.2936**
SFBD Food Web	128	2,106	0.0724	0.0700	0.0860	0.0566	0.0113	**0.1654**	0.0725	0.1215
E.Coli	418	519	0.0243	0.0088	0.0009	0.0012	0.0003	0.0118	0.0278	**0.0334**
Web Edu	3,031	6,474	0.4267	0.2273	0.1928	0.2675	0.0103	0.0121	0.4247	**0.5032**
Advogato	5,155	39,285	0.1329	0.1214	**0.1548**	0.0122	0.0023	0.0502	0.1390	**0.1539**
PGP	10,680	24,316	0.3110	0.2591	0.3136	0.0824	0.0107	0.0212	0.2831	**0.3434**
EAT	23,219	304,938	0.0665	0.0652	**0.0911**	0.0016	0.0003	0.0126	0.0413	0.0651
AS CAIDA 2007 11 05	26,475	53,381	0.0480	0.0444	0.0488	0.0004	0.0000	0.0327	0.0350	**0.0534**
Func-Func	46,027	106,510	0.0546	0.0395	0.0007	0.0060	0.0000	0.0497	0.0437	**0.0799**
Livemocha	104,103	2,193,083	0.0119	0.0116	0.0262	0.0002	0.0000	0.0152	0.0113	**0.0307**
Amazon	334,863	925,872	0.1677	0.1272	0.1636	0.0888	0.0345	0.0004	0.1584	**0.1850**
Twitter Follows	404,719	713,319	0.0017	0.0008	0.0017	0.0003	0.0000	0.0013	0.0021	**0.0025**

#### Comparison against local methods on the CAIDA AS relationships dataset

**Figure 4:**
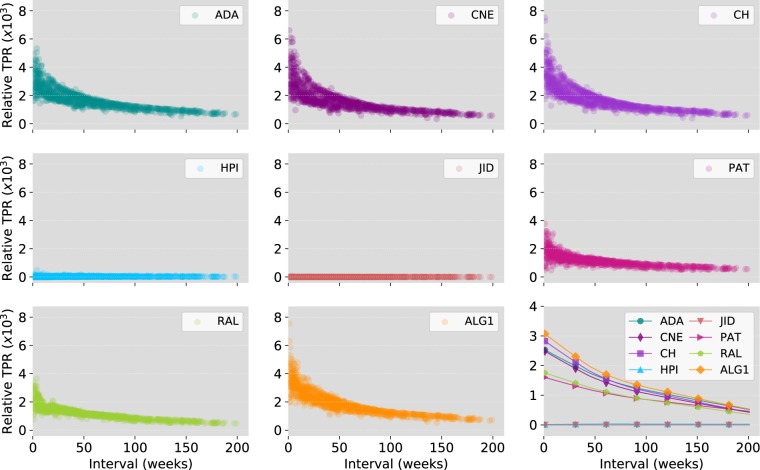
Comparison of Algorithm 1 with local link prediction methods on the CAIDA AS relationships networks. For each algorithm, we scatter plot relative top-precisions vs. the prediction interval in weeks. The line plots are obtained by smoothing the data using LOWESS (Locally Weighted Scatterplot Smoothing) with 80% of the data used for estimating each $y$-value.

In this experiment, we compare the performance of the proposed algorithm against local methods on the CAIDA AS relationships dataset, which contains 122 CAIDA AS graphs collected from January 2004 to November 2007. The data was collected periodically, once a month during 2004 and 2005, then weekly in 2006 and 2007. Each of the 122 networks contains the full AS graph derived from a set of RouteViews BGP table snapshots. As a pre-processing step, we removed the network collected on 17/09/2007 which is clearly and outlier as its size is abnormally small compared to the other networks (see Table 3 of SI). Our goal is to assess the strength of the algorithms at predicting the future evolution of the network given its present state. For this, we consider all couples of networks ordered according to the date of collection and use the earlier network to predict the later one. Newly appeared edges, that is, those present in the second network but not in the first one, are used as a test set. We run the algorithms on all 7,260 network couples and compute the top-precision obtained by each algorithm on every couple. To account for the systematic increase in top-precision due to the increase of the test set size, we compute the *relative top-precision* which we define as the top-precision of the algorithm divided by the top-precision of a random link predictor (hence, the random predictor has, by definition, a relative top-precision of 1). In Fig. 4, we scatter plot relative top-precision against the interval separating the collection dates of the two networks measured in weeks. We also plot the smoothed version of the data (line plot) obtained using LOWESS (Locally Weighted Scatterplot Smoothing) with 80% of the data used for estimating each top precision value. The results show that Algorithm 1 gives the best predictions for all interval durations followed by CH for short to medium predication intervals and ADA for longer intervals. As shown by the statistical significance tests in Table 1, Algorithm 1 gives the highest top-precision overall followed by CH then ADA.

#### Comparison against local methods on synthetic networks

To gain a better understanding of the performance of the proposed algorithm relatively to neighbourhood-based methods and the effects of network topology on its predictive power, we conduct an experimental evaluation using synthetic networks generated using the following well-known complex networks models:

- Nonuniform popularity-similarity optimization (nPSO) networks: Several studies on complex networks have suggested the existence of hidden geometrical spaces behind their topologies^6,7,25,31–33^. The popularity-similarity optimization (PSO) model^33^ suggests that complex networks can be embedded into a hyperbolic space, where nodes are mapped to angular and radial coordinates. Nodes with high similarity are mapped to close angular coordinates, and popular nodes, characterized by high degrees, are assigned to lower radial coordinates compared to other nodes. The nonuniform popularity-similarity optimization (nPSO) model^34^ is a variation of the popularity-similarity-optimization (PSO) model in which the angular coordinates are sampled from a Gaussian mixture distribution with $C$ components instead of a uniform distribution; allowing thus the formation of community structures, each corresponding to a component of the Gaussian mixture. A parameter called temperature ($T$) controls the mixing of the communities; more links are generated between nodes that are far in the disk when the temperature $T$ is increased, leading thus to a lower clustering coefficient. In addition, half the average node degree $k$ and the exponent of the power-law degree distribution $\gamma$ are provided as an input parameter to the model.
- Watts-Strogatz networks: The Watts-Strogatz model^35^ allows the generation of small-world networks, that is, networks with small diameter. It constructs a ring lattice of $n$ nodes, each of them is connected to its $k$ previous and $k$ next neighbours, then each link is rewired with a probability $p$ to a uniformly selected node from the remaining nodes if the link is not already present.
- Barabàsi-Albert networks: Real networks are usually characterized by a power law degree distribution that distinguishes them from random networks. The Barabàsi-Albert model^36,37^ is one of the first models that allows the generation of scale-free networks by adding gradually nodes with $k$ undirected edges and attaching the other end of each of them to another node in the network with a probability proportional to the degree of that node.

**Figure 5:**
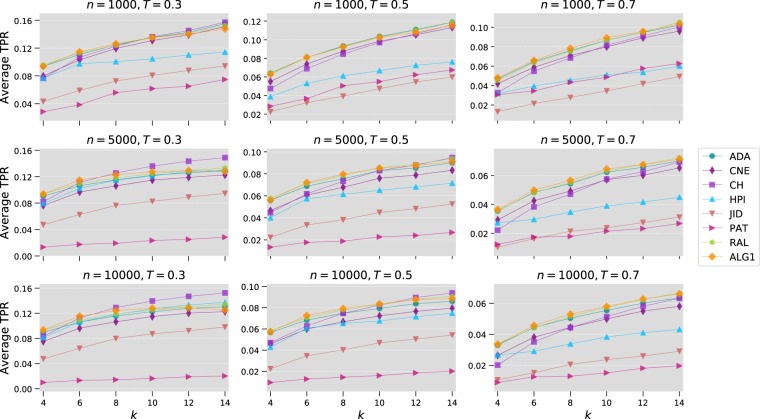
Comparison of Algorithm 1 with local link prediction methods on nPSO networks. We report the average top-precision obtained using 100 randomly generate networks for every combination of $n,k$ and $T$ (here $\gamma$ is fixed to 3).

For the experiment on nPSO networks, we generate networks with 8 communities $(C=8)$ having the following parameters: $n=1000,5000,10000$, $k=4,6,8,10,12,14$, $T=0.3,0.5,0.7$ and $\gamma =3$. This value assigned to $\gamma$ is commonly encountered in real networks. For each of the 54 combinations of these parameters, we generate 100 samples randomly. Each sample is then used to test the link prediction methods by removing 10% of the edges and using them as a test set. Figure 5 shows the average top-precision results obtained with different values of the network parameters. All methods show a decrease in performance as the networks become larger and less clustered, that is when $T$ increases. On the other hand, the performance increases when the density of the networks increases (larger $k$). The proposed algorithm gives consistently good results when the networks are sparse ($k=4,6$). For denser networks, CH gives the best results for $T=0.3$, that is, in highly clustered networks, but when clustering becomes weaker at $T=0.5$ or 0.7, Algorithm 1 and RAL take the lead. The latter two methods also produce the best results overall as can be seen from the statistical tests results of Table 1.

**Figure 6:**
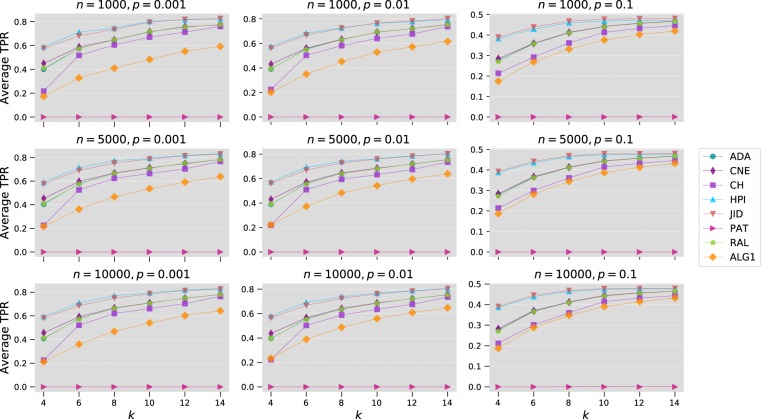
Comparison of Algorithm 1 with local link prediction methods on Watts-Strogatz networks. In the plot, we show the average top-precision obtained using 100 randomly generate networks for every combination of $n,k$ and $p$.

A similar procedure is followed for tests on the Watts-Strogatz networks. The following parameters are used: $n=1000,5000,10000$, $k=4,6,8,10,12,14$ and $p=0.001,0.01,0.1$. Results of average top-precision are shown in Fig. 6, whereas Table 1 includes the associated statistical significance tests. Clearly, popularity-based methods such as the proposed algorithm perform very poorly in this type of networks. This is due to the fact that despite exhibiting the small-world property, Watts-Strogatz networks are not scale-free networks, a property shared by many, if not the majority, of real networks. Consequently, methods that rely on node degree to score links fail to accurately predict the topology of this type of networks. Interestingly, the rank results in Watts-Strogatz networks also show an almost reversal of the algorithms’ ranks compared to the other types of networks. Methods such as JID and HPI index, which performed poorly elsewhere, produce here the best results. The fact that the topology of real networks rarely follow the Watts-Strogatz model, however, limits the successful use of these methods in practical situations.

**Figure 7:**
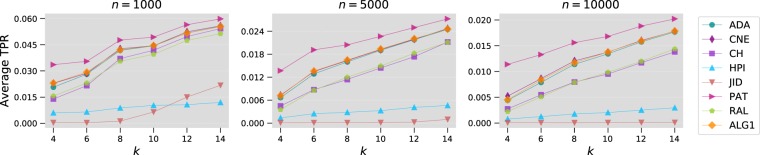
Comparison of Algorithm 1 with local link prediction methods on Barabàsi-Albert networks. In the plot, we report the top-precision averaged over 100 randomly generated networks as function of $k$.

For Barabàsi-Albert networks, we generate 100 random samples of networks having a combination of the following parameters: $n=1000,5000,10000$ and $k=4,6,8,10,12,14$. Average top-precision results are shown in Fig. 7, and Table 1 includes the associated statistical significance tests. Since Barabàsi-Albert networks evolve according to the preferential attachment principle, whereby new connections are more likely to be made with nodes having already high degrees, the preferential attachment method (PAT) gives the best results followed immediately by CNE and Algorithm 1.

#### Effect of network topology on the performance of the proposed method

**Figure 8:**
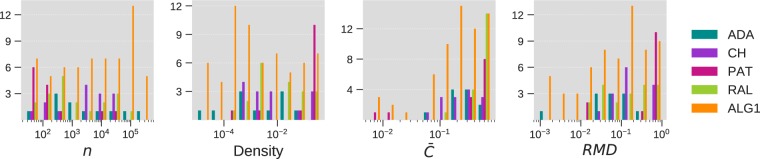
Effect of network topology on the performance of the proposed method. Using the real networks employed in the experimental evaluation, we plot the histograms of number of nodes, $n$, density, average clustering coefficient ($\bar{C}$) and relative max-degree (RMD) grouped according to the best performing method (only the five most frequent methods are considered).

To understand the effect of network topology on Algorithm 1, we use the results obtained on the real networks employed in the previous experiments to plot the grouped histograms shown in Fig. 8. Four topological properties are considered: the order of the network ($n$), its density, its average clustering coefficient and its relative max-degree defined as $RMD={\kappa }_{\max }/(n-1)$. The networks are grouped according to the best performing method based on top-precision. Clear distinctions between the five prediction algorithms can be observed from the figure. ADA performs comparatively well in low to medium dense networks that are highly clustered. CH and RAL are effective in highly clustered networks with medium to high density. PAT produces good results in networks with high $RMD$, which are typically small and highly dense networks (such as food web networks) but scales poorly to medium and large networks. Finally, the results show that the proposed algorithm maintains a high predictive power over a wide spectrum of networks. It produces particularity strong results in highly sparse and weakly clustered networks, a type of networks that pauses clear difficulties to neighbourhood-based methods (such as ADA, CH and RAL) due to the absence of a rich local structure. Algorithm 1 remedies to this lack of information by taking into account the popularity and the estimated similarity of the nodes to predict connections. On the other hand, when the network is dense and clustered, the algorithm relies on the local attraction term, ${\eta }_{ij}$, to take advantage of the available information and produce accurate predictions.

#### Time performance

**Figure 9:**
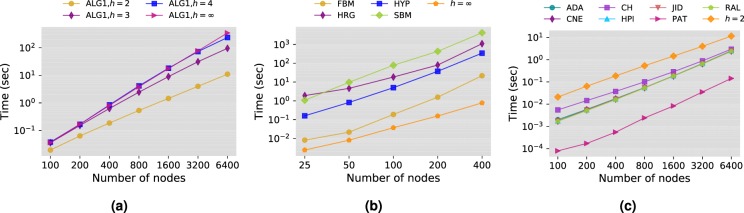
Time performance of the proposed algorithm. The evaluation is performed using randomly generated networks using Barabàsi-Albert model with $k=8$. The time reported is the average time required by the algorithms to compute the score of all non-existing links. In (**a**), we report the running time of Algorithm 1 using different values of the horizon cut-off $h$. Each data point is the average over 10 runs on 100 different networks. In (**b**), we report the time performance of Algorithm 1 with $h=\infty$ against global prediction methods. Each data point is the average over 100 different networks. In (**c**), we report the time performance of Algorithm 1 with $h=2$ against local methods. Each data point is the average over 10 runs on 100 different networks.

To compare the time performance of the proposed method to the other methods, we randomly generate several Barabàsi-Albert networks with varying number of nodes and use them as input for the link prediction algorithms. We used the implementation provided by the authors of the four methods HRG, SBM, FBM and HYP, and our implementation for the remaining methods (see SI for details on the implementation). Figure 9 shows the running time of the algorithms in seconds as a function of the number of nodes in the network. The plot in Fig. 9(a) shows the effect of the horizon cut-off $h$ on the running time of Algorithm 1. Clearly, using the limit $h=2$ produces considerable gain in performance compared to other values, even small ones such as 3 or 4. The results of Fig. 9(b) demonstrate that even with the extreme limit $h=\infty$, that is running full Dijkstra, the proposed method is several orders of magnitude faster than the existing global methods. Finally, Fig. 9(c) shows the comparison against local methods. We can see that although Algorithm 1 requires more time to finish due to running Dijkstra, it has a growth rate that is comparable to the other methods and maintains a reasonably low computational cost even for large networks.

## Discussion

The proposed link prediction method falls into the class of global, parameter-free similarity-popularity-based link predictors, in which we assume that network topology is governed by three factors: the popularity of the nodes, their similarity, and the attraction caused by their local neighbourhood. In our approach, popularity and local attraction are computed directly from the network topology and are factored out through a weight map that is used to estimate the similarity between non-adjacent nodes via shortest path distances. The proposed algorithm is non-iterative and, therefore, does not suffer from convergence issues as do many other link prediction methods, especially Monte Carlo type ones. The experimental analysis shows that the proposed approach achieves high predictive power at a much lower computational cost compared to existing global methods. In comparison to local methods, the proposed algorithm offers more accurate results at a low additional computational cost. Indeed, as demonstrated experimentally, the algorithm can in fact be used on very large networks having hundreds of thousands of nodes. By combining different sources of information to predict links, namely popularity, similarity and local attraction, the proposed algorithm maintains a high predictive over a wide spectrum of network types and sizes. It is particularly powerful in highly sparse and weakly clustered networks, a type of networks that proves challenging for local methods.

The computationally intensive part of the proposed algorithm is solving the shortest path problem for all links to be predicted. We have seen that imposing a horizon cut-off when running Dijkstra’s algorithm can considerably reduce the computation time without loss of precision. It is also theoretically possible to relax the condition of having exact distances and settle for an approximation of these distances. This especially true given that the edge lengths are heuristically assigned. It would be interesting to investigate the use of graph embedding methods^38^ to approximate shortest path distances and reduce the computational cost when working with large networks. Finally, depending on the type of network at hand, it is conceivable that properties other than popularity and other forms of local attractions may affect the likelihood of connection between nodes. A generalization of the present method can in principle be designed for such cases.

## Methods

To demonstrate the effectiveness of the proposed approach, we use simulated networks as well as 106 real networks having different sizes and originating from different domains: social, biological, technological and informational^39–54^. Description and statistics on some important structural properties of these networks are presented in Table 2 of SI. Statistics on the CAIDA AS relationships networks are reported in Table 3 of SI. The reported performance results are calculated as averages over several test runs. In each trial, a randomly selected set of edges is removed from the network and used as part of the test set along with the original set of negative links. The network composed of the remaining edges is presented to the prediction algorithms.

In addition to the comparison with global link prediction methods discussed earlier, the proposed method is compared to several local topological ranking methods which are described in what follows (the description of the remaining local methods used in the evaluation is included in the SI):

- Adamic-Adar index (ADA) assigns to each couple $(i,j)$ the score ${s}_{ij}$ defined by:
  8 $${s}_{ij}=\sum _{k\in {\Gamma }_{ij}}\frac{1}{{\rm{\log }}\,({\kappa }_{k})}$$
  where ${\Gamma }_{ij}$ is the set of nodes adjacent to both $i$ and $j$, and ${\kappa }_{k}$ is the degree of node $k$.
- Common neighbours (CNE) assigns the score ${s}_{ij}=| {\Gamma }_{ij}|$.
- Cannistraci-Hebb index (CH) assigns the score ${s}_{ij}={\sum }_{k\in {\Gamma }_{ij}}| {\Gamma }_{k}\cap {\Gamma }_{ij}| /{\kappa }_{k}$, where ${\Gamma }_{k}$ is the set of nodes adjacent to node $k$.
- Hub promoted index (HPI) assigns the score ${s}_{ij}=| {\Gamma }_{ij}| /\min ({\kappa }_{i},{\kappa }_{j})$.
- Jackard index (JID) assigns the score ${s}_{ij}=| {\Gamma }_{ij}| /\left({\kappa }_{i}+{\kappa }_{j}-| {\Gamma }_{ij}| \right)$.
- Preferential attachment index (PAT) assigns a score which depends only on the degrees of $i$ and $j$ making it a pure popularity method:
  9 $${s}_{ij}={\kappa }_{i}{\kappa }_{j}.$$
- Resource allocation index (RAL) assigns the score:
  10 $${s}_{ij}=\sum _{k\in {\Gamma }_{ij}}\frac{1}{{\kappa }_{k}}.$$

## Supplementary information

Supplementary Information.

## Data Availability

No datasets were generated or analysed during the current study.
