# Supplementary material for: A Scalable Similarity-Popularity Link Prediction Method
Source: Sci Rep. 2020 Apr 14;10:6394. doi: 10.1038/s41598-020-62636-1 (PMC7156691; doi:10.1038/s41598-020-62636-1)
Supplement: Supplementary file 1 — Supplementary Information. [file 41598_2020_62636_MOESM1_ESM.pdf]

A Scalable Similarity-Popularity Link Prediction Method (Supporting Information)

Said Kerrache[1*], **Ruwayda Alharbi**[1], **and Hafida Benhidour**[1]

[1]King Saud University, College of Computer and Information Sciences, Riyadh, 11543, KSA
[*]skerrache@ksu.edu.sa

This document contains supporting information to the main article. It is organized as follows: we start by give some details about the implementation of the proposed method and the methods used in the evaluation, we then describe the networks used in the experiments. The subsequent section includes detailed and additional results of the performance of Algorithm 1 on real networks. We conclude with a comparison against two previously proposed weighting rules: the repulsion-attraction pre-weighting rules[1].

1 Implementation

All link prediction methods used in the experimental evaluation are implemented in C++, and all experiments are conducted in a Linux environment:

- We implemented in C++ the proposed algorithms and all the topological similarity methods used in the experiments. The description of the topological ranking methods used in the experimental evaluation is given in Table 1.
- For HRG[9], we used the code provided by the authors[1].
- For SBM[10], we used the C code provided by the authors[2]. This algorithm has a single parameter which is the number of iterations, and in our case we set it to the default value 10000.
- For FBM[11], we translated the Matlab code provided by the authors[3] into C++. This algorithm has a single parameter which is the number of iterations, and in our case we set it to the default value 50.
- For HyperMap (HYP)[12, 13], we used the code provided by the authors[4]. This algorithm has five parameters which are all set according to the guidelines provided by the authors:

    1. m: represents the average number of nodes to which new nodes connect and is set to the minimum node degree.
    2. L: represents the average number of nodes to which old nodes connect and is set to $L = (\langle k \rangle - 2m)/2$, where $\langle k \rangle$ is the average node degree.
    3. γ: is the exponent of the power-law degree distribution. In our case, it is estimated using *plfit*, a C++ implementation of Clauset, Shalizi and Newman[14] method for fitting power law distributions written by Tamas Nepusz[5].
    4. T: controls the average clustering and is set to 0.8.
    5. $\zeta = \sqrt{-K}$ where K is the curvature of the hyperbolic plane. This is set to 1.

[1]HRG implementation is available at http://tuvalu.santafe.edu/~aaronc/hierarchy/hrg_20120527_predictHRG_v1.0.4.zip.

[2]Available at http://seeslab.info/media/filer_public/eb/ae/ebaee03f-a53a-430f-a4a1-6b713d36e91e/rgraph-2.0.1.tar.gz

[3]The code was obtained directly from the main author.

[4]The code is available at http://www.cut.ac.cy/eecei/staff/f.papadopoulos/?languageId=2.

[5]Implementation available at http://tuvalu.santafe.edu/~aaronc/powerlaws/

- For CH[15] (previously named CRA[16,17]), we translated the Matlab code published by the authors[6] into C++. This algorithm has no parameters.

2 Data

Several real networks have been used in the experimental evaluation of the proposed method. These networks are publicly available through different data repositories[2–8]. Table 2 contains the description and some important structural properties of these networks. Statistics on the CAIDA AS relationships networks are shown in Table 3. Notice that, whereas some networks like Zakary's Karate Club or DNA citation are small in size, networks such as Amazon, Twitter Follows or US Patents contain hundreds of thousands of nodes. These large networks obviously cannot be used to test the performance of methods such as SBM or HRG due to their high computational requirements, and are therefore only used to only evaluate scalable methods. Each data point in the plots included in the main paper is the average result of several test runs. In each test run, 10% of the edges are randomly removed from the network. The number of tests is fixed to 1000 for small networks (having less than 1000 nodes) and 100 for networks with higher nodes count.

3 Detailed results

This section contains detailed, per-network results of the experiments reported in the paper. Table 4, 5 and 6 show respectively the AUPR, AUROC and top-precision obtained using different values of the horizon cutoff on small networks. Table 7 shows the top-precision obtained using different values of the horizon cutoff on large networks. Table 8 shows the results per network for the comparison of Algorithm 1 against global link prediction methods on 18 small network. Table 12 shows the comparison of the proposed algorithm against local methods on 40 small networks. Results on 40 large networks are shown in Table 10, whereas Table 11 shows the results on 26 very large networks.

4 Further results

This section contains additional experimental results. Namely, we report the results obtained with small networks for all 12 methods we are comparing against. Table 12 contains the average top-precision obtained for each network as well as the average significant rank, whereas Table 13 shows the associated statistical significance tests.

5 Comparison with the repulsion-attraction pre-weighting rules

In[1], authors present fast algorithms for embedding large graphs in the hyperbolic circle. They propose the repulsion-attraction rule (RA), that uses the neighbourhood topological information to estimate the distances between the connected nodes. These distances are used to pre-weight the network links. The repulsive force acts between adjacent nodes with high degrees and low number of common neighbours because they represent hubs and should be geometrically far. On the contrary, the attractive force acts between adjacent nodes with high number of common neighbours because they most likely are similar and should be geometrically close. Formally, given the network's adjacency matrix A, the weight w of the link (i,j) can be computed using one of the following formulas:

$$w_{ij}^{RA1} = \frac{\kappa_i + \kappa_j + \kappa_i \kappa_j}{1 + \Gamma_{ij}} \qquad (if A_{ij} = 1), \tag{1}$$

$$w_{ij}^{RA2} = \frac{1 + e_i + e_j + e_i e_j}{1 + \Gamma_{ij}} \qquad (if A_{ij} = 1), \tag{2}$$

where κ_i is the degree of node i; e_i is the external degree of node i (links neither to Γ_{ij} nor to j); Γ_{ij} common neighbors of nodes i and j.

We compare the performance obtained when using RA1 and RA2 to pre-weight the networks links instead of the rule used in Algorithm 1. We use 38 real networks to conduct the comparison. Table 14 contains the average top-precision obtained for each network as well as the average significant rank. Table 15 shows the associated statistical significance tests. The results show that although the RA1 and RA2 rules perform better than a random predictor, meaning they do possess a certain predictive power, they rarely outperform the proposed rule. Furthermore, the difference is statistically significant and is considerable in most networks.

[6]The code is available at https://sites.google.com/site/carlovittoriocannistraci/5-datasets-and-matlab-code/car-based-indices-and-local-community-paradigm

References

1. Muscoloni, A., Thomas, J. M., Ciucci, S., Bianconi, G. & Cannistraci, C. V. Machine learning meets complex networks via coalescent embedding in the hyperbolic space. *Nat. Commun.* **8**, 1615 (2017).
2. Leskovec, J. & Krevl, A. SNAP Datasets: Stanford large network dataset collection. http://snap.stanford.edu/data (2014).
3. Zitnik, M., Sosič, R., Maheshwari, S. & Leskovec, J. BioSNAP Datasets: Stanford biomedical network dataset collection. http://snap.stanford.edu/biodata (2018).
4. Kunegis, J. Konect: The koblenz network collection. In *Proceedings of the 22Nd International Conference on World Wide Web*, WWW '13 Companion, 1343–1350 (ACM, New York, NY, USA, 2013). URL http://konect.uni-koblenz.de/networks.
5. Batagelj, V. & Mrvar, A. Pajek datasets. http://vlado.fmf.uni-lj.si/pub/networks/data (2006).
6. Rossi, R. A. & Ahmed, N. K. The network data repository with interactive graph analytics and visualization. In *AAAI* (2015). URL http://networkrepository.com.
7. Zafarani, R. & Liu, H. Social computing data repository at ASU (2009). URL http://socialcomputing.asu.edu.
8. Szklarczyk, D. *et al.* STRING v11: protein–protein association networks with increased coverage, supporting functional discovery in genome-wide experimental datasets. *Nucleic Acids Res.* **47**, D607–D613 (2018).
9. Clauset, A., Moore, C. & Newman, M. E. Hierarchical structure and the prediction of missing links in networks. *Nat.* **453**, 98–101 (2008).
10. Guimerà, R. & Sales-Pardo, M. Missing and spurious interactions and the reconstruction of complex networks. *Proc. Natl. Acad. Sci.* **106**, 22073–22078 (2009).
11. Liu, Z., He, J.-L., Kapoor, K. & Srivastava, J. Correlations between community structure and link formation in complex networks. *PloS one* **8** (2013).
12. Papadopoulos, F., Kitsak, M., Serrano, M. Á., Boguná, M. & Krioukov, D. Popularity versus similarity in growing networks. *Nat.* **489**, 537–540 (2012).
13. Papadopoulos, F., Psomas, C. & Krioukov, D. Network mapping by replaying hyperbolic growth. *IEEE/ACM Transactions on Netw. (TON)* **23**, 198–211 (2015).
14. Clauset, A., Shalizi, C. R. & Newman, M. E. Power-law distributions in empirical data. *SIAM review* **51**, 661–703 (2009).
15. Muscoloni, A. & Cannistraci, C. V. Local-ring network automata and the impact of hyperbolic geometry in complex network link-prediction (2017). Preprint at http://arXiv:1707.09496 [physics.soc-ph].
16. Cannistraci, C. V., Alanis-Lobato, G. & Ravasi, T. From link-prediction in brain connectomes and protein interactomes to the local-community-paradigm in complex networks. *Sci. reports* **3** (2013).
17. Daminelli, S., Thomas, J. M., Durán, C. & Cannistraci, C. V. Common neighbours and the local-community-paradigm for topological link prediction in bipartite networks. *New J. Phys.* **17**, 113037 (2015).
18. Almunia, J., Basterretxea, G., Aristegui, J. & Ulanowicz, R. Benthic-pelagic switching in a coastal subtropical lagoon. *Estuarine, Coast. Shelf Sci.* **49**, 363 – 384 (1999).
19. Zachary, W. W. An information flow model for conflict and fission in small groups. *J. anthropological research* **33**, 452–473 (1977).
20. Monaco, M. & Ulanowicz, R. Comparative ecosystem trophic structure of three u.s. mid-atlantic estuaries. *Mar. Ecol. Prog. Ser.* **161**, 239–254. (1997).
21. Hummon, N. P. & Dereian, P. Connectivity in a citation network: The development of dna theory. *Soc. Networks* **11**, 39–63 (1989).
22. Hagy, J. D. *Eutrophication, hypoxia and trophic transfer efficiency in Chesapeake Bay*. Ph.D. thesis, University of Maryland at College Park (USA) (2002).
23. Baird, D., Luczkovich, J. & Christian, R. Assessment of spatial and temporal variability in ecosystem attributes of the st marks national wildlife refuge, apalachee bay, florida. *Estuarine, Coast. Shelf Sci.* **47**, 329 – 349 (1998).
24. Guimerà, R., Mossa, S., Turtschi, A. & Amaral, L. A. N. The worldwide air transportation network: Anomalous centrality, community structure, and cities' global roles. *Proc. Natl. Acad. Sci. United States Am.* **102**, 7794–9 (2005).

25. Krebs, V. E. Mapping networks of terrorist cells. *Connect.* **24**, 43–52 (2002).

26. Hayes, B. Connecting the dots. can the tools of graph theory and social-network studies unravel the next big plot? *Am. Sci.* **94**, 400–404 (2006).

27. Ulanowicz, R. E., Heymans, J. J. & Egnotovich, M. S. Analysis of trophic dynamics in south florida ecosystems, fy 99: The graminoid ecosystem. Tech. Rep., University of Maryland System Chesapeake Biological Laboratory (2000).

28. Knuth, D. E. *The Stanford GraphBase: A Platform for Combinatorial Computing* (Addison-Wesley Professional, Reading, MA, 1994), 1st edn.

29. Isella, L. *et al.* What's in a crowd? analysis of face-to-face behavioral networks. *J. theoretical biology* **271**, 166–180 (2011).

30. Hummon, N., Doreian, P. & Freeman, L. Analyzing the structure of the centrality-productivity literature created between 1948 and 1979. *Knowl.* **11**, 459–480 (1990).

31. Michalski, R., Palus, S. & Kazienko, P. Matching organizational structure and social network extracted from email communication. In *Business Information Systems*, 197–206 (Springer, 2011).

32. Gleiser, P. & Danon, L. Community Structure in Jazz. *Adv. Complex Syst.* **6**, 565–573 (2003).

33. Stehlé, J. *et al.* High-resolution measurements of face-to-face contact patterns in a primary school. *PLOS ONE* **6**, 1–13 (2011). URL https://doi.org/10.1371/journal.pone.0023176. DOI 10.1371/journal.pone.0023176.

34. Freeman, L., Webster, C. & Kirke, D. Exploring social structure using dynamic three-dimensional color images. *Soc. Networks* **20**, 109–118 (1998).

35. Coleman, J., Katz, E. & Menzel, H. The diffusion of an innovation among physicians. *Sociom.* **20**, 253–270 (1957).

36. Chaintreau, A. *et al.* Impact of human mobility on opportunistic forwarding algorithms. *IEEE Transactions on Mob. Comput.* **6**, 606–620 (2007). DOI 10.1109/TMC.2007.1060.

37. Watts, D. J. & Strogatz, S. H. Collective dynamics of 'small-world'networks. *nature* **393**, 440–442 (1998).

38. White, J. G., Southgate, E., Thomson, J. N. & Brenner, S. The Structure of the Nervous System of the Nematode Caenorhabditis elegans. *Philos. Transactions Royal Soc. Lond. B: Biol. Sci.* **314**, 1–340 (1986).

39. Modha, D. S. & Singh, R. Network architecture of the long-distance pathways in the macaque brain. *Proc. Natl. Acad. Sci.* **107**, 13485–13490 (2010).

40. Isella, L. *et al.* What's in a crowd? analysis of face-to-face behavioral networks. *J. Theor. Biol.* **271**, 166 – 180 (2011).

41. Shen-Orr, S. S., Milo, R., Mangan, S. & Alon, U. Network motifs in the transcriptional regulation network of escherichia coli. *Nat. genetics* **31**, 64–68 (2002).

42. Jeong, H., Tombor, B., Albert, R., Oltvai, Z. N. & Barabási, A.-L. The large-scale organization of metabolic networks. *Nat.* **407**, 651–654 (2000).

43. Brenner, S. The genetics of caenorhabditis elegans. *Genet.* **77**, 71–94 (1974).

44. Adamic, L. A. & Glance, N. The political blogosphere and the 2004 us election: divided they blog. In *Proceedings of the 3rd international workshop on Link discovery*, 36–43 (ACM, 2005).

45. S.Heymannm, S. & Palmier, J. Source code structure of a java program (2008).

46. Roget, P. *Roget's Thesaurus of English Words and Phrases* (Prabhat Prakashan, 1962).

47. Knuth, D. E. *The Stanford GraphBase: A Platform for Combinatorial Computing* (ACM, New York, NY, USA, 1993).

48. Guimerà, R., Danon, L., Díaz-Guilera, A., Giralt, F. & Arenas, A. Self-similar community structure in a network of human interactions. *Phys. Rev. E* **68**, 065103 (2003).

49. Goh, K.-I. *et al.* The human disease network. *Proc. Natl. Acad. Sci.* **104**, 8685–8690 (2007).

50. Newman, M. E. Finding community structure in networks using the eigenvectors of matrices. *Phys. review E* **74**, 036104 (2006).

51. Opsahl, T. & Panzarasa, P. Clustering in weighted networks. *Soc. networks* **31**, 155–163 (2009).

52. Bu, D. *et al.* Topological structure analysis of the protein–protein interaction network in budding yeast. *Nucleic Acids Res.* **31**, 2443–2450 (2003).

53. Mainas, E. *The Analysis of Criminal and Terrorist Organisations as Social Network Structures*. Master's thesis, Institute of Criminal Justice Studies, University of Portsmouth, UK (2009).

54. Reitz, J. M. Odlis: Online dictionary of library and information science (2002).

55. Gleich, D., Zhukov, L. & Berkhin, P. Fast parallel pagerank: A linear system approach. *Yahoo! Res. Tech. Rep. YRL-2004-038* **13**, 22 (2004).

56. De Nooy, W., Mrvar, A. & Batagelj, V. *Exploratory social network analysis with Pajek*, vol. 27 (Cambridge University Press, 2011).

57. Castillo, C., Chellapilla, K. & Denoyer, L. Web spam challenge 2008. In *Proceedings of the 4th International Workshop on Adversarial Information Retrieval on the Web (AIRWeb)* (2008).

58. Massa, P., Salvetti, M. & Tomasoni, D. Bowling alone and trust decline in social network sites. In *Proc. Int. Conf. Dependable, Autonomic and Secure Computing*, 658–663 (2009).

59. Leskovec, J., Kleinberg, J. & Faloutsos, C. Graph evolution: Densification and shrinking diameters. *ACM Trans. Knowl. Discov. from Data* **1**, 1–40 (2007).

60. Kumar, S., Spezzano, F., Subrahmanian, V. & Faloutsos, C. Edge weight prediction in weighted signed networks. In *Data Mining (ICDM), 2016 IEEE 16th International Conference on*, 221–230 (IEEE, 2016).

61. Kumar, S. *et al.* Rev2: Fraudulent user prediction in rating platforms. In *Proceedings of the Eleventh ACM International Conference on Web Search and Data Mining*, 333–341 (ACM, 2018).

62. Ripeanu, M., Foster, I. & Iamnitchi, A. Mapping the gnutella network: Properties of large-scale peer-to-peer systems and implications for system design. *IEEE Internet Comput. J.* **6** (2002).

63. Leskovec, J., Huttenlocher, D. & Kleinberg, J. Signed networks in social media. In *Proceedings of the SIGCHI Conference on Human Factors in Computing Systems*, CHI '10, 1361–1370 (ACM, New York, NY, USA, 2010).

64. Leskovec, J., Huttenlocher, D. & Kleinberg, J. Predicting positive and negative links in online social networks. In *Proceedings of the 19th International Conference on World Wide Web*, WWW '10, 641–650 (ACM, New York, NY, USA, 2010).

65. Norlen, K., Lucas, G., Gebbie, M. & Chuang, J. Eva: Extraction, visualization and analysis of the telecommunications and media ownership network. In *Proceedings of International Telecommunications Society 14th Biennial Conference, Seoul Korea*, 27–129 (2002).

66. Boguñá, M., Pastor-Satorras, R., Díaz-Guilera, A. & Arenas, A. Models of social networks based on social distance attachment. *Phys. review E* **70**, 056122 (2004).

67. Leskovec, J., Kleinberg, J. & Faloutsos, C. Graphs over time: densification laws, shrinking diameters and possible explanations. In *Proceedings of the eleventh ACM SIGKDD international conference on Knowledge discovery in data mining*, KDD '05, 177–187 (ACM, New York, NY, USA, 2005).

68. Boldi, P. & Vigna, S. The WebGraph framework I: Compression techniques. In *Proc. of the Thirteenth International World Wide Web Conference (WWW 2004)*, 595–601 (ACM Press, Manhattan, USA, 2004).

69. Boldi, P., Rosa, M., Santini, M. & Vigna, S. Layered label propagation: A multiresolution coordinate-free ordering for compressing social networks. In Srinivasan, S. *et al.* (eds.) *Proceedings of the 20th international conference on World Wide Web*, 587–596 (ACM Press, 2011).

70. Corman, S. R., Kuhn, T., Mcphee, R. D. & Dooley, K. J. Studying complex discursive systems. *Hum. Commun. Res.* **28**, 157–206 (2002).

71. Paranjape, A., Benson, A. R. & Leskovec, J. Motifs in temporal networks. In *Proceedings of the Tenth ACM International Conference on Web Search and Data Mining*, WSDM '17, 601–610 (ACM, New York, NY, USA, 2017).

72. Boldi, P., Codenotti, B., Santini, M. & Vigna, S. UbiCrawler: A scalable fully distributed web crawler. *Software: Pract. & Exp.* **34**, 711–726 (2004).

73. Kiss, G. R., Armstrong, C., Milroy, R. & Piper, J. An associative thesaurus of English and its computer analysis. In Aitkin, A. J., Bailey, R. W. & Hamilton-Smith, N. (eds.) *The computer and literary studies* (University Press, Edinburgh, UK, 1973).

74. Leskovec, J. & Mcauley, J. J. Learning to discover social circles in ego networks. In Pereira, F., Burges, C. J. C., Bottou, L. & Weinberger, K. Q. (eds.) *Advances in Neural Information Processing Systems 25*, 539–547 (Curran Associates, Inc., 2012).

75. De Choudhury, M., Sundaram, H., John, A. & Seligmann, D. D. Social synchrony: Predicting mimicry of user actions in online social media. In *International Conference on Computational Science and Engineering*, vol. 4, 151–158 (IEEE, 2009).

76. Leskovec, J., Lang, K. J., Dasgupta, A. & Mahoney, M. W. Community structure in large networks: Natural cluster sizes and the absence of large well-defined clusters. *Internet Math.* **6**, 29–123 (2009).

77. Klimt, B. & Yang, Y. Introducing the enron corpus. In *CEAS* (2004).

78. Rozemberczki, B., Davies, R., Sarkar, R. & Sutton, C. A. GEMSEC: graph embedding with self clustering. *CoRR* **abs/1802.03997** (2018). 1802.03997.

79. Ashburner, M. *et al.* Gene ontology: tool for the unification of biology. *Nat. Genet.* **25**, 25–29 (2000).

80. Cho, E., Myers, S. A. & Leskovec, J. Friendship and mobility: User movement in location-based social networks. In *Proceedings of the 17th ACM SIGKDD International Conference on Knowledge Discovery and Data Mining*, KDD '11, 1082–1090 (ACM, New York, NY, USA, 2011).

81. Viswanath, B., Mislove, A., Cha, M. & Gummadi, K. P. On the evolution of user interaction in facebook. In *Proceedings of the 2Nd ACM Workshop on Online Social Networks*, WOSN '09, 37–42 (ACM, New York, NY, USA, 2009).

82. Richardson, M., Agrawal, R. & Domingos, P. Trust management for the semantic web. In *Proceedings of the Second International Conference on Semantic Web Conference*, LNCS-ISWC'03, 351–368 (Springer-Verlag, Berlin, Heidelberg, 2003).

83. Yahoo! Webscope. Dataset: ydata-ymessenger-user-communication-pattern-v1_0 (2008). URL http://research.yahoo.com/Academic_Relations.

84. Hall, B., Jaffe, A. & Trajtenberg, M. The nber patent citation data file: Lessons, insights and methodological tools. NBER Working Papers 8498, National Bureau of Economic Research, Inc (2001).

85. Batagelj, V. Efficient algorithms for citation network analysis. *CoRR* (2003).

86. Leskovec, J., Adamic, L. A. & Huberman, B. A. The dynamics of viral marketing. *ACM Trans. Web* **1** (2007).

87. Yang, J. & Leskovec, J. Defining and evaluating network communities based on ground-truth. *Knowl. Inf. Syst.* **42**, 181–213 (2015).

88. Gleich, D. F. & Rossi, R. A. A dynamical system for pagerank with time-dependent teleportation. *Internet Math.* **10**, 188–217 (2014).

Table 1. Description of the topological ranking methods used in the experimental evaluation.

Method	Score assigned to the couple (i, j)
Adamic-Adar index (ADA)	$s_{ij} = \sum_{k \in \Gamma_{ij}} \frac{1}{\log(\kappa_k)}$, where Γ_{ij} is the set of nodes adjacent to both i and j, and κ_k is the degree of node k.
Common neighbours (CNE)	$s_{ij} = \lvert\Gamma_{ij}\rvert$.
Cannistraci-Hebb index (CH)	$s_{ij} = \sum_{k \in \Gamma_{ij}} \frac{\lvert\Gamma_k \cap \Gamma_{ij}\rvert}{\kappa_k}$, where Γ_k is the set of nodes adjacent to node k.
Hub depromoted index (HDI)	$s_{ij} = \frac{\lvert\Gamma_{ij}\rvert}{\max(\kappa_i, \kappa_j)}$.
Hub promoted index (HPI)	$s_{ij} = \frac{\lvert\Gamma_{ij}\rvert}{\min(\kappa_i, \kappa_j)}$.
Jackard index (JID)	$s_{ij} = \frac{\lvert\Gamma_{ij}\rvert}{\kappa_i + \kappa_j - \lvert\Gamma_{ij}\rvert}$.
Leicht-Holme-Newman index (LHN)	$s_{ij} = \frac{\lvert\Gamma_{ij}\rvert}{\kappa_i \kappa_j}$.
Preferential attachment index (PAT)	$s_{ij} = \kappa_i \kappa_j$.
Resource allocation index (RAL)	$s_{ij} = \sum_{k \in \Gamma_{ij}} \frac{1}{\kappa_k}$.
Salton index (SAI)	$s_{ij} = \frac{\lvert\Gamma_{ij}\rvert}{\sqrt{\kappa_i \kappa_j}}$.
Sorensen index (SOI)	$s_{ij} = \frac{\lvert\Gamma_{ij}\rvert}{\kappa_i + \kappa_j}$.

Table 2. Description and statistics on the structural characteristics of the networks used in the experimental analysis. Columns n and m represent the number of nodes and links in the network, respectively. Columns $\langle k \rangle$ and C denote the average degree and the average clustering coefficient. All networks are considered undirected.

Network	Description	n	m	$\langle k \rangle$	C
Maspalomas[18]	Food web of Maspalomas coastal lagoon (Canary Islands). This dataset is available at http://vlado.fmf.uni-lj.si/pub/networks/data/bio/foodweb/Maspalomas.paj.	24	77	6.42	0.45
Zakary's Karate Club[19]	A social network that represents friendships between members of a karate club at an American university. The data was collected in the 1970s by Wayne Zachary and is available at http://konect.uni-koblenz.de/networks/ucidata-zachary	34	78	4.59	0.57
Narragan[20]	Food web of the Narragansett estuary. This dataset is available at http://vlado.fmf.uni-lj.si/pub/networks/data/bio/foodweb/Narragan.paj.	35	204	11.66	0.62
DNA Citation CC[21]	The main connected component of the network DNA Citation (see below).	35	59	3.37	0.18
DNA Citation[21]	A citation network of DNA research literature. The vertices represent research papers, and the edges represent the citations. This dataset is available at http://vlado.fmf.uni-lj.si/pub/networks/Data/cite/default.htm.	39	61	3.13	0.16
ChesLower[22]	Food web of Lower Chesapeake Bay in summer. This dataset is available at http://vlado.fmf.uni-lj.si/pub/networks/data/bio/foodweb/ChesLower.paj.	37	167	9.03	0.49
ChesMiddle[22]	Food web of Middle Chesapeake Bay in summer. This dataset is available at http://vlado.fmf.uni-lj.si/pub/networks/data/bio/foodweb/ChesMiddle.paj.	37	198	10.70	0.60
ChesUpper[22]	Food web of Upper Chesapeake Bay in summer. This dataset is available at http://vlado.fmf.uni-lj.si/pub/networks/data/bio/foodweb/ChesUpper.paj.	37	199	10.76	0.59
StMarks[23]	Food web of St Marks National Wildlife Refuge. This dataset is available at http://vlado.fmf.uni-lj.si/pub/networks/data/bio/foodweb/Maspalomas.paj.	54	350	12.96	0.41
Japan Air	Air transportation network between Japanese cities. This network is extracted from the World Transport network[24], available at http://seeslab.info/media/filer_public/63/97/63979ddc-a625-42f9-9d3d-8fdb4d6ce0b0/airports.zip.	56	183	6.54	0.58
Terrorist[25]	Terrorist associations for 9/11 attacks, where vertices are individuals associated with the 9/11 terrorist attacks, and edges indicate social associations. The dataset is available at http://tuvalu.santafe.edu/~aaronc/hierarchy/terrorists.zip.	62	152	4.90	0.49
Terrorist Train Bombing[26]	A social network that represents contacts between a set of terrorists who were involved in the train bombing of Madrid on March 11, 2004. This dataset is available at http://konect.uni-koblenz.de/networks/moreno_train.	64	243	7.59	0.62
Everglades[27]	Food web of Everglades graminoid marshes in the wet season. This dataset is available at http://vlado.fmf.uni-lj.si/pub/networks/data/bio/foodweb/Everglades.paj.	69	880	25.51	0.55
Cypress Dry[27]	Food web of Cypress (Florida) in the dry season. This dataset is available at http://vlado.fmf.uni-lj.si/pub/networks/data/bio/foodweb/cypdry.paj.	71	618	17.41	0.50
Cypress Wet[27]	Food web of Cypress (Florida) in the wet season. This dataset is available at http://vlado.fmf.uni-lj.si/pub/networks/data/bio/foodweb/cypwet.paj.	71	612	17.24	0.50
Les Miserables[28]	Network of coappearance of characters in Victor Hugo's novel "Les Miserables". The data is available at http://www-personal.umich.edu/~mejn/netdata/lesmis.zip.	77	254	6.6	0.74
Polbooks	In this network vertices represent books about US politics sold by the online bookseller Amazon.com. Edges represent frequent co-purchasing of books by the same buyers. The network was compiled by V. Krebs and is unpublished, but can found at http://www-personal.umich.edu/~mejn/netdata/.	105	441	8.40	0.49
ACM2009 Contacts[29]	Network of face-to-face contacts of the attendees of the ACM Conference on Hypertext and Hypermedia 2009. The dataset is available at http://konect.uni-koblenz.de/networks/sociopatterns-hypertext.	113	2,196	38.87	0.53
Centrality Literature[30]	Citation network among papers on network centrality from 1948 to 1979. The dataset is available at http://vlado.fmf.uni-lj.si/pub/networks/data/GD/a01.zip.	118	613	10.39	0.37

Table 2. (continued)

Network	Description	*n*	*m*	$\langle k \rangle$	*C*
SFBW Food Web[27]	South Florida food web in the wet season. This data consists of network of carbon exchange as they normally occur in the ecosystems of South Florida. Vertices represent major components of the ecosystem, and edges represent the transfers of material or energy between the components. The dataset is available at http://vlado.fmf.uni-lj.si/pub/networks/data/bio/foodweb/baywet.paj.	128	2,075	32.42	0.33
SFBD Food Web[27]	South Florida food web in the dry season. This data consists of network of carbon exchange as they normally occur in the ecosystems of South Florida. Vertices represent major components of the ecosystem, and edges represent the transfers of material or energy between the components. The dataset is available at http://vlado.fmf.uni-lj.si/pub/networks/data/bio/foodweb/baydry.paj.	128	2,106	32.91	0.33
Manufacturing e-mail[31]	Email communication network between employees of a mid-sized manufacturing company. The dataset is available at https://www.ii.pwr.edu.pl/~michalski/datasets/manufacturing.tar.gz.	167	3,250	38.92	0.59
Jazz[32]	A network of collaborations between Jazz musicians. The dataset is available at http://deim.urv.cat/~alexandre.arenas/data/xarxes/jazz.zip.	198	2,742	27.7	0.31
School[33]	A network of face-to-face proximity between students and teachers in a primary school. The dataset is available at http://www.sociopatterns.org/datasets/primary-school-cumulative-networks (day 1).	236	5,899	49.99	0.50
Residence Hall[34]	A symmetrized version of a social network representing friendship relationship between residents living in a residence hall at the Australian National University campus. The dataset is available at http://moreno.ss.uci.edu/data.html#oz.	217	1,839	16.95	0.36
Physicians[35]	The network captures innovation spread among physicians in the towns in Illinois, Peoria, Bloomington, Quincy and Galesburg. The data was collected in 1966. A node represents a physician and an edge between two physicians shows that the left physician told that the right physician is his friend or that he turns to the right physician if he needs advice or is interested in a discussion. This dataset is available at http://konect.uni-koblenz.de/networks/moreno_innovation.	241	923	7.66	0.31
GD 01	Citation network among papers of the Graph Drawing (GD) context from GD94 to GD2000. The dataset is available at http://vlado.fmf.uni-lj.si/pub/networks/data/GD/a01.zip.	259	640	4.94	0.23
Haggle Contact[36]	A network representing contacts between persons measured by carried wireless devices. The dataset is available at http://konect.uni-koblenz.de/networks/contact.	274	2,124	15.50	0.63
C. Elegans Neural[37]	A symmetrized version of a directed, weighted network representing the neural network of the worm C. Elegans. The network is compiled by D. Watts and S. Strogatz and made available at http://cdg.columbia.edu/cdg/datasets. Original experimental data was taken from[38].	297	2,148	14.46	0.29
US Air 97	North American Transportation Atlas Data (NORTAD). The data is available at http://vlado.fmf.uni-lj.si/pub/networks/data/map/USAir97.net.	332	2,126	12.81	0.63
Macaque Neural[39]	The network represents the macaque brain network. Each node corresponds to a brain region and each link represents a long-distance connection between two brain regions. The dataset is available at http://www.pnas.org/content/107/30/13485.full (the network is named Macaque_LongDistance_Network_connectivity.edgelist by the authors).	360	5,208	28.93	0.42
Infectious[40]	Face-to-face interaction between visitors of the exhibition INFECTIOUS: STAY AWAY in 2009 at the Science Gallery in Dublin. A link indicates that a face-to-face interaction took place for more than 20 seconds. The dataset is available at http://konect.uni-koblenz.de/networks/sociopatterns-infectious.	410	2,765	13.49	0.46
E.Coli[41]	A biological network representing the transcriptional regulation of Escherichia coli. In this network the vertices are operons, and each edge is directed from an operon that encodes a transcription factor to an operon that it directly regulates. The dataset is available at http://vlado.fmf.uni-lj.si/pub/networks/data/GD/GD.htm.	418	519	2.48	0.09
Erdos 971	Paul Erdös is a mathematician researcher. This data is the 1971 version of Erdös' co-authorship network where vertices represent the authors. An edge exists between two vertices if they have published at least one paper together. The dataset is available at https://sparse.tamu.edu/MM/Pajek/Erdos971.tar.gz.	433	1,314	6.07	0.28
Erdos 981	Paul Erdös is a mathematician researcher. This data is the 1981 version of Erdös' co-authorship network where vertices represent the authors. An edge exists between two vertices if they have published at least one paper together. The dataset is available at https://sparse.tamu.edu/MM/Pajek/Erdos981.tar.gp{9.5cm}z.	433	1,314	6.07	0.28
C. Elegans Metabolic[42]	A metabolic network of the worm C. Elegans[43]. The vertices represent metabolites (e.g., proteins), and edges represent interactions between them. The data is available at http://konect.uni-koblenz.de/networks/arenas-meta	453	2,038	9.00	0.65

Table 2. (continued)

Network	Description	n	m	$\langle k\rangle$	C
Erdos 991	Paul Erdös is a mathematician researcher. This data is the 1991 version of Erdös' co-authorship network where vertices represent the authors. An edge exists between two vertices if they have published at least one paper together. The dataset is available at https://sparse.tamu.edu/MM/Pajek/Erdos991.tar.gz.	454	1,417	6.24	0.29
Political Blogs[44]	A network of hyperlinks between web blogs on the Untied States politics. The data is available at http://networkrepository.com/web-polblogs.php.	643	2,280	7.09	0.23
Codeminer[45]	This network represents the call-graph of Java program. It consists of 724 vertices representing packages, classes, fields and methods. Edges between the vertices represent calls (from method to method) and containments (packages to classes, between classes, and classes to methods). The dataset is available at https://github.com/gephi/gephi.github.io/tree/master/datasets.	724	1,015	2.80	0.15
CPAN Authors	This network represents the relationships between the developers of the Perl language. This snapshot was created by Linkfluence in July 2009. The vertices represent developers, whereas an edge indicates that the two developers use the same Perl module. This data is available at https://gephi.org/datasets/cpan-authors.gexf.zip.	839	2,112	5.03	0.35
Roget[46,47]	A network of cross-references between the 1022 categories in Roget's Thesaurus (the data is part of Stanford GraphBase[47]). The dataset is available at http://vlado.fmf.uni-lj.si/pub/networks/data/dic/roget/Roget.htms.	1,010	3,648	7.22	0.15
Email[48]	A symmetrized version of the email communication network at the University Rovira i Virgili (Tarragona, Spain). Nodes represent users, and edges indicate the at least one email was sent from one user to another. The dataset is available at http://deim.urv.cat/~alexandre.arenas/data/welcome.htm.	1,133	5,451	9.62	0.22
FAA[4]	Network of preferred routes between US airports constructed from USA's FAA (Federal Aviation Administration) National Flight Data Center (NFDC). The dataset can be found at http://konect.uni-koblenz.de/networks/maayan-faa.	1,226	2,408	3.93	0.07
Diseasome[49]	A network of disorders and disease genes linked by known disorder–gene associations. The data can be found at http://gephi.org/datasets/diseasome.gexf.zip.	1,419	2,738	3.86	0.49
Network Science[50]	A co-authorship network of researchers in the field of network science. The data was compiled by M. Newman in May 2006. The data can be found at http://www-personal.umich.edu/~mejn/netdata/netscience.zip.	1,461	2,742	3.75	0.69
Java	The Java Compile-Time Dependency graph consists of nodes representing Java classes and directed edges representing compile-time dependencies between two classes. The dataset is available at http://vlado.fmf.uni-lj.si/pub/networks/data/GD/GD.htm.	1,538	7,817	10.17	0.40
Oclinks[51]	A symmetrized version of an online weighted social network created from an online community at the University of California, Irvine, which covers the period from April to October 2004. Nodes represent students that sent or received at least one message, and edges represent the exchange of messages between them. The network, weighted by number of messages, is available at https://toreopsahl.com/datasets/#online_social_network.	1,899	13,838	14.57	0.11
Yeast[52]	A network of protien-protien interaction in budding yeast. The data is available at http://vlado.fmf.uni-lj.si/pub/networks/data/bio/Yeast/Yeast.htm.	2,284	6,646	5.82	0.13
Criminal[53]	A network of phone calls bep9.5cmtween the members of a drug trafficking group. The dataset is available at https://sites.google.com/site/ucinetsoftware/datasets/mainaseuropoldatasets.	2,749	2,952	2.15	0.07
ODLIS[54]	Hypertext network based on the Online Dictionary of Library and Information Science (ODLIS). An edge from term X to term Y exists in the network if in the ODLIS dictionary the term Y is used to describe the meaning of term X. The dataset is available at http://vlado.fmf.uni-lj.si/pub/networks/data/dic/odlis/Odlis.htm.	2,900	16,377	11.29	0.30
Web Edu[55]	A web network.The data is available at http://networkrepository.com/web-edu.php.	3,031	6,474	4.27	0.56
World Transport[24]	A worldwide air transportation network, where vertices represent cities and edges indicate the existence of a flight connecting two cities. The data is available at http://seeslab.info/media/filer_public/63/97/63979ddc-a625-42f9-9d3d-8fdb4d6ce0b0/airports.zip.	3,618	14,142	7.82	0.50
Web EPA[56]	A web network of pages linking to www.epa.gov. Network available at http://networkrepository.com/web-EPA.php.	4,271	8,909	4.17	0.07
Terror[53]	Terrorist network collected during a counter-terrorism intelligence operation in the EU (nodes are persons). The dataset is available at https://sites.google.com/site/ucinetsoftware/datasets/mainaseuropoldatasets.	4,275	6,531	3.06	0.17
Spam[57]	A web network. Network available at http://networkrepository.com/web-spam.php.	4,767	37,375	15.68	0.29

Table 2. (continued)

Network	Description	n	m	$\langle k \rangle$	C
Power[37]	An undirected, unweighted network representing the topology of the Western States Power Grid of the United States. Network available at http://www-personal.umich.edu/~mejn/netdata/.	4,941	6,594	2.67	0.08
Advogato[58]	A symmetrized version of the Advogato trust network, where nodes represent users and edges represent trust relationships. Network available at http://konect.uni-koblenz.de/networks/advogato.	5,155	39,285	15.24	0.25
GR[59]	The network of collaboration between authors in arXiv's general relativity and quantum cosmology section. Nodes represent authors, and an edge represents a co-authored publication. Network available at https://snap.stanford.edu/data/ca-GrQc.html.	5,241	14,484	5.53	0.53
Bitcoin[60,61]	A symmetrized version of the trust network between users trading in Bitcoin on the platform Bitcoin OTC. The network is weighted and signed, but weights are ignored and edges with negative weight are removed. The data is available at https://snap.stanford.edu/data/soc-sign-bitcoin-otc.html.	5,881	21,492	7.31	0.18
Gnutella 08[59,62]	A snapshot of the Gnutella peer-to-peer file sharing network on August 8, 2002. The vertices represent hosts, and edges represent connections between the hosts. The dataset can be found at https://snap.stanford.edu/data/p2p-Gnutella08.txt.gz.	6,301	20,777	6.59	0.01
Erdos 02	Paul Erdös is a mathematician researcher. This data is the 2002 version of Erdös' co-authorship network where vertices represent the authors. An edge exists between two vertices if they have published at least one paper together. The dataset is available at http://vlado.fmf.uni-lj.si/pub/networks/data/Erdos/Erdos02.net.	6,927	11,850	3.42	0.12
WikiVote[63,64]	Network of users votes on Wikipedia. An edge between two users, means that one user voted for the other. Network available at https://snap.stanford.edu/data/wiki-Vote.html.	7,115	100,762	28.32	0.14
EVA[65]	Company ownership network, where nodes are companies and edges represent ownership relationships (isolated nodes are removed). The data is available at http://vlado.fmf.uni-lj.si/pub/networks/data/econ/Eva/Eva.htm.	7,253	6,711	1.85	0.01
Lederberg	Network of articles written by and citing the American molecular biologist J. Lederberg. The data is available at http://vlado.fmf.uni-lj.si/pub/networks/data/cite/Lederberg.zip	8,324	41,539	9.98	0.31
Hero	A social networks of the super heroes from the Marvel universe. The data is constructed by Cesc Rosselló, Ricardo Alberich, and Joe Miro from the University of the Balearic Islands and is available at https://gephi.org/datasets/hero-social-network.gephi.	10,469	178,115	34.03	0.46
PGP[66]	A social network of users who exchange secure information using Pretty Good Privacy (PGP) algorithm. The network is available at http://deim.urv.cat/~alexandre.arenas/data/welcome.htm.	10,680	24,316	4.55	0.27
Oregon[67]	Autonomous Systems (AS) peering network inferred from Oregon route-views on May 26, 2001.The data is available at https://snap.stanford.edu/data/oregon1_010526.txt.gz.	11,174	23,409	4.19	0.30
Indochina 2004[68,69]	A web network.The data is available at http://networkrepository.com/web_indochina_2004.php.	11,358	47,606	8.38	0.71
Reuters 9 11[70]	Network of word association in all stories released by Reuters regarding the September 11 attacks during the 66 days following event. The nodes are words; an edge between two words means these two words appear in the same sentence. Network available at http://vlado.fmf.uni-lj.si/pub/networks/data/CRA/terror.htm.	13,314	148,038	22.24	0.37
Youtube[7]	A friendship network on Youtube. Network available at http://socialcomputing.asu.edu/pages/datasets.	13,723	76,764	11.19	0.14
Mathoverflow C2A[71]	Network of comment-to-answer interactions between users on Mathoverflow. Network available at https://snap.stanford.edu/data/sx-mathoverflow.html.	13,778	71,234	10.34	0.18
Web Base 2001[69,72]	A web network.The data is available at http://networkrepository.com/web-webbase-2001.php.	16,062	25,593	3.19	0.22
AS Internet	A snapshot of the structure of the Internet at the level of autonomous systems (ASs), reconstructed from BGP tables provided by the University of Oregon Route Views Project. The data was collected by Mark Newman using data for July 22, 2006 and is available at http://www-personal.umich.edu/~mejn/netdata/.	17,258	45,702	5.30	0.38
Astro[59]	The network of collaboration between authors in arXiv's astrophysics (astro-ph) section. Nodes represent authors, and an edge represents a co-authored publication. Network available at http://konect.uni-koblenz.de/networks/ca-AstroPh.	18,771	198,050	21.10	0.63

Table 2. (continued)

Network	Description	n	m	$\langle k \rangle$	C
Gnutella 25[59,62]	A snapshot of the Gnutella peer-to-peer file sharing network on August 25, 2002. The vertices represent hosts, and edges represent connections between the hosts. The dataset can be found at https://snap.stanford.edu/data/p2p-Gnutella08.txt.gz.	22,687	54,705	4.82	0.01
Cond Mat[59]	The network of collaboration between authors in arXiv's condensed matter section. Nodes represent authors, and an edge represents a co-authored publication. Network available at https://snap.stanford.edu/data/ca-CondMat.html.	23,133	93,439	8.08	0.63
EAT[73]	The Edinburgh Associative Thesaurus is a network of word association as collected from subjects. Network available at http://vlado.fmf.uni-lj.si/pub/networks/data/dic/eat/Eat.htm.	23,219	304,938	26.27	0.10
G Plus[74]	A network of circles from Google plus. Data available at https://snap.stanford.edu/data/ego-Gplus.html.	23,628	39,194	3.32	0.17
AS CAIDA[59]	The Internet network at the autonomous systems level from the CAIDA project. The data was collected in 2007. Network available at http://konect.uni-koblenz.de/networks/as-caida20071105.	26,475	53,381	4.03	0.21
HepTh Cit[67]	A symmterized version of the citation network in arXiv's high energy physics theory section. Nodes represent papers, and edges represent citations. Network available at https://snap.stanford.edu/data/cit-HepTh.html.	27,769	352,285	25.37	0.31
Digg Reply[75]	A reply network on the news social platform Digg. Nodes represent users, and an edge indicates a reply of one user to an other. The data is available at http://networkrepository.com/ia_digg_reply.php.	30,360	85,155	5.61	0.01
HepPh Cit[67]	A symmterized version of the citation network in arXiv's high energy physics - phenomenology section. Nodes represent papers, and edges represent citations. Network available at https://snap.stanford.edu/data/cit-HepPh.html.	34,546	420,877	24.37	0.28
Enron[76,77]	Enron email communication network. Nodes are email addresses; an edge means that at least one email has been exchanged between the two corresponding addresses. The data is available at https://snap.stanford.edu/data/email-Enron.html.	36,692	183,831	10.02	0.50
Deezer RO[78]	Friendship network among users of the music streaming service Deezer collected in November 2017. This is the network of users from Romania. The data is available at https://snap.stanford.edu/data/gemsec-Deezer.html.	41,773	125,826	6.02	0.09
Func-Func[79]	Network of biological functions. Data available at https://snap.stanford.edu/biodata/datasets/10026/10026-FF-Miner.html.	46,027	106,510	4.63	0.10
Deezer HU[78]	Friendship network among users of the music streaming service Deezer collected in November 2017. This is the network of users from Hungary. The data is available at https://snap.stanford.edu/data/gemsec-Deezer.html.	47,538	222,887	9.38	0.17
Facebook Artist[78]	A network of Facebook artist pages connected by mutual likes. Data available at https://snap.stanford.edu/data/gemsec-Facebook.html.	50,515	819,090	32.43	0.14
Deezer HR[78]	Friendship network among users of the music streaming service Deezer collected in November 2017. This is the network of users from Croatia. The data is available at https://snap.stanford.edu/data/gemsec-Deezer.html.	54,573	498,202	18.26	0.14
Brightkite[80]	Friendship network on the social platform Brightkite. Network available at https://snap.stanford.edu/data/loc-Brightkite.html.	58,228	214,078	7.35	0.17
Gnutella 31[59,62]	A snapshot of the Gnutella peer-to-peer file sharing network on August 31, 2002. The vertices represent hosts, and edges represent connections between the hosts. The dataset can be found at https://snap.stanford.edu/data/p2p-Gnutella31.txt.gz.	62,586	147,892	4.73	0.01
Facebook[81]	A Facebook friendship network. Data available at http://konect.uni-koblenz.de/networks/facebook-wosn-links.	63,392	816,886	25.77	0.22
Wordnet	A network where nodes are words and the existence of an edge means that the two words are related. The data is available at http://vlado.fmf.uni-lj.si/pub/networks/data/dic/Wordnet/Wordnet.htm	73,753	234,024	6.35	0.46
Epinions[82]	Trust network among users of the consumer review site Epinions.com. Network available at https://snap.stanford.edu/data/soc-Epinions1.html.	75,879	405,740	10.69	0.14
Slashdot 09 02[76]	Network of friend/foe relationships between the users of Slashdot. The data was collected in February 2009. Network available at https://snap.stanford.edu/data/soc-Slashdot0902.html.	82,168	504,230	12.27	0.06
Yahoo IM[83]	Network of sample Yahoo! Messenger communication events. The data was collected 28 during days starting from April 1, 2008. Network available at https://webscope.sandbox.yahoo.com/catalog.php?datatype=g.	100,001	587,964	11.76	0.20
Superuser C2A[71]	Network of comment-to-answer interactions between users on the stack exchange web site http://superuser.com. Network available at https://snap.stanford.edu/data/sx-superuser.html.	100,391	263,973	5.26	0.06

Table 2. (continued)

Network	Description	n	m	$\langle k \rangle$	C
Livemocha[7]	Friendship network crawled from www.livemocha.com in December 2010 by Xia Hu (Ben) (xiahu@asu.edu).. Network available at http://socialcomputing.asu.edu/datasets/Livemocha.	104,103	2,193,083	42.13	0.05
SK 2005[69,72]	A partial web crawling of .sk domain in 2005.The data is available at http://networkrepository.com/web-sk-2005.php.	121,422	334,419	5.51	0.23
Internet[5]	Network of Internet routers. The data is available at https://sparse.tamu.edu/Pajek/internet.	124,651	193,620	3.11	0.06
Askubuntu A2Q[71]	Network of answer-to-question interactions between users on the stack exchange web site http://askubuntu.com. Network available at https://snap.stanford.edu/data/sx-askubuntu.html.	134,035	246,781	3.68	0.02
Douban[7]	Friendship network among users of douban.com, a Chinese website for recommending movies, books and music. The data is available at http://socialcomputing.asu.edu/datasets/Douban.	154,908	327,162	4.22	0.02
Gowalla[80]	Friendship network on the social platform Gowalla. Network available at https://snap.stanford.edu/data/loc-Gowalla.html.	196,591	950,327	9.67	0.24
US Patents[84,85]	Partial citation network of US patents. The data is available at https://www.cise.ufl.edu/research/sparse/matrices/Pajek/patents_main.html.	240,547	560,943	4.66	0.05
Amazon 03 02[86]	Amazon product co-purchasing network on March 02 2003. Nodes represent products; an edge means that the two products have been co-purchased. Network available at https://snap.stanford.edu/data/amazon0302.html.	262,111	899,792	6.87	0.42
Amazon[87]	Amazon product co-purchasing network. Nodes represent products; an edge means that the two products have been co-purchased. Network available at https://snap.stanford.edu/data/com-Amazon.html.	334,863	925,872	5.53	0.40
Twitter Follows[88]	A network of Twitter users connected by the follow relationship. Network available at http://networkrepository.com/soc-twitter-follows.php.	404,719	713,319	3.53	0.01

Table 3. Description and statistics on the structural characteristics of the networks of the CAIDA AS relationships dataset. Columns $|V|$ and $|E|$ represent the number of nodes and links in the network, respectively. Columns $\langle k \rangle$ and C denote the average degree and the average clustering coefficient. All networks are considered undirected.

Network collection date	$\|V\|$	$\|E\|$	$\langle k \rangle$	C
2004 01 05	16,301	32,955	4.04	0.23
2004 02 02	16,493	33,372	4.05	0.23
2004 03 01	16,655	33,340	4.00	0.23
2004 04 05	16,874	34,335	4.07	0.24
2004 05 03	17,160	35,013	4.08	0.24
2004 06 07	17,306	35,547	4.11	0.24
2004 07 05	17,509	35,829	4.09	0.23
2004 08 02	17,655	36,070	4.09	0.24
2004 09 06	17,848	37,172	4.17	0.24
2004 10 04	18,100	37,497	4.14	0.25
2004 11 01	18,278	37,559	4.11	0.25
2004 12 06	18,501	38,265	4.14	0.24
2005 01 03	18,740	38,501	4.11	0.25
2005 02 07	18,911	37,991	4.02	0.24
2005 03 07	19,090	38,562	4.04	0.24
2005 04 04	19,267	38,930	4.04	0.24
2005 05 02	19,489	39,859	4.09	0.24
2005 06 06	19,720	40,198	4.08	0.24
2005 07 04	19,846	40,485	4.08	0.25
2005 08 01	20,037	40,474	4.04	0.25
2005 09 05	20,344	40,941	4.02	0.25
2005 10 03	20,513	41,069	4.00	0.24
2005 11 07	20,731	41,762	4.03	0.24
2005 12 05	20,889	41,820	4.00	0.24
2006 01 02	21,202	42,925	4.05	0.24
2006 01 09	21,157	42,734	4.04	0.23
2006 01 16	21,232	42,872	4.04	0.24
2006 01 23	21,245	42,481	4.00	0.23
2006 01 30	21,339	43,283	4.06	0.24
2006 02 06	21,343	43,425	4.07	0.24
2006 02 13	21,402	43,616	4.08	0.24
2006 02 20	21,525	43,969	4.09	0.24
2006 02 27	21,548	43,892	4.07	0.23
2006 03 06	21,598	44,245	4.10	0.23
2006 03 13	21,583	43,780	4.06	0.23
2006 03 20	21,658	44,308	4.09	0.23
2006 03 27	21,672	44,327	4.09	0.24
2006 04 03	21,754	44,322	4.07	0.23
2006 04 10	21,734	43,902	4.04	0.23
2006 04 17	21,861	44,829	4.10	0.23
2006 04 24	21,885	44,472	4.06	0.23

Table 3. (continued)

Network collection date	$\|V\|$	$\|E\|$	$\langle k \rangle$	C
2006 05 01	21,901	44,588	4.07	0.22
2006 05 08	22,030	44,839	4.07	0.22
2006 05 15	22,072	44,780	4.06	0.22
2006 05 22	22,086	44,576	4.04	0.22
2006 05 29	22,191	45,086	4.06	0.22
2006 06 05	22,205	45,001	4.05	0.22
2006 06 12	22,270	45,410	4.08	0.22
2006 06 19	22,332	45,392	4.07	0.23
2006 06 26	22,317	45,196	4.05	0.22
2006 07 03	22,456	45,050	4.01	0.22
2006 07 10	22,442	45,550	4.06	0.22
2006 07 17	22,461	45,148	4.02	0.22
2006 07 24	22,602	46,085	4.08	0.22
2006 07 31	22,640	45,738	4.04	0.22
2006 08 07	22,706	46,413	4.09	0.22
2006 08 14	22,735	46,167	4.06	0.22
2006 08 21	22,749	46,085	4.05	0.22
2006 08 28	22,848	46,578	4.08	0.21
2006 09 04	22,918	47,090	4.11	0.22
2006 09 11	22,965	47,193	4.11	0.22
2006 09 18	23,001	46,864	4.07	0.22
2006 09 25	23,149	47,368	4.09	0.22
2006 10 02	23,126	46,779	4.05	0.22
2006 10 09	23,195	47,534	4.10	0.22
2006 10 16	23,247	46,606	4.01	0.22
2006 10 23	23,350	47,242	4.05	0.22
2006 10 30	23,353	46,655	4.00	0.22
2006 11 06	23,390	46,095	3.94	0.21
2006 11 13	23,072	39,342	3.41	0.15
2006 11 20	23,601	48,006	4.07	0.22
2006 11 27	23,634	48,202	4.08	0.22
2006 12 04	23,663	47,777	4.04	0.22
2006 12 11	23,783	48,648	4.09	0.22
2006 12 18	23,884	48,746	4.08	0.22
2006 12 25	23,918	49,089	4.10	0.22
2007 01 01	24,013	49,332	4.11	0.22
2007 01 08	24,056	49,391	4.11	0.22
2007 01 15	24,018	49,196	4.10	0.22
2007 01 22	24,078	49,056	4.07	0.22
2007 01 29	20,906	42,994	4.11	0.21
2007 02 05	24,142	49,200	4.08	0.22
2007 02 12	24,191	49,225	4.07	0.22
2007 02 19	24,267	48,986	4.04	0.21

Table 3. (continued)

Network collection date	$\lvert V \rvert$	$\lvert E \rvert$	$\langle k \rangle$	C
2007 02 26	24,297	49,583	4.08	0.22
2007 03 05	24,454	49,830	4.08	0.21
2007 03 12	24,491	49,826	4.07	0.21
2007 03 19	24,542	50,183	4.09	0.22
2007 03 26	24,610	50,738	4.12	0.22
2007 04 02	24,649	50,462	4.09	0.22
2007 04 09	24,776	50,827	4.10	0.22
2007 04 16	24,801	50,881	4.10	0.22
2007 04 23	24,810	49,958	4.03	0.22
2007 04 30	24,969	51,238	4.10	0.22
2007 05 07	24,942	51,145	4.10	0.22
2007 05 14	25,090	51,178	4.08	0.21
2007 05 21	25,056	51,106	4.08	0.21
2007 05 28	25,158	51,234	4.07	0.21
2007 06 04	25,265	51,755	4.10	0.21
2007 06 11	25,314	51,444	4.06	0.21
2007 06 18	25,304	51,510	4.07	0.21
2007 06 25	25,352	52,048	4.11	0.21
2007 07 02	25,474	52,317	4.11	0.21
2007 07 09	25,526	52,412	4.11	0.21
2007 07 16	25,477	52,363	4.11	0.21
2007 07 23	25,579	52,546	4.11	0.21
2007 07 30	25,696	52,666	4.10	0.21
2007 08 06	25,697	52,762	4.11	0.21
2007 08 13	25,826	52,401	4.06	0.21
2007 08 20	25,741	52,423	4.07	0.21
2007 08 27	25,800	52,383	4.06	0.21
2007 09 03	25,988	52,365	4.03	0.20
2007 09 10	26,022	52,691	4.05	0.20
2007 09 17[7]	8,020	18,203	4.54	0.27
2007 09 24	26,139	53,156	4.07	0.21
2007 10 01	26,184	53,289	4.07	0.21
2007 10 08	26,242	53,174	4.05	0.21
2007 10 15	26,258	53,601	4.08	0.21
2007 10 22	26,369	53,231	4.04	0.21
2007 10 29	26,377	53,255	4.04	0.21
2007 11 05	26,475	53,381	4.03	0.21
2007 11 12	26,389	52,861	4.01	0.21

[7] This networks is clearly an outlier as its size is abnormally small compared to the other networks. Consequently, it has been removed from the tests.

Table 4. Effect of the horizon cut-off h on the performance of Algorithm 1 in small networks (AUPR). We report the AUPR for different values of h averaged over 1000 test runs where 10% of the edges are removed while keeping the network connected. For every network, the results having the best significant rank with $p = 0.05$ are shown in bold. The last row shows the average significant rank over all networks (the lower the better). The columns n and m contain the number of nodes and edges in the network.

Network	n	m	$h=2$	$h=3$	$h=4$	$h=5$	$h=6$	$h=7$	$h=8$	$h=9$	$h=\infty$
ACM2009 Contacts	113	2,196	**0.233**	**0.233**	**0.233**	**0.233**	**0.233**	**0.233**	**0.233**	**0.233**	**0.233**
C.Elegans Metabolic	453	2,038	**0.142**	**0.142**	**0.142**	**0.142**	**0.142**	**0.142**	**0.142**	**0.142**	**0.142**
C.Elegans Neural	297	2,148	**0.058**	**0.058**	**0.058**	**0.058**	**0.058**	**0.058**	**0.058**	**0.058**	**0.058**
CPAN Authors	37	167	**0.036**	**0.037**	**0.037**	**0.037**	**0.037**	**0.037**	**0.037**	**0.037**	**0.037**
Centrality Literature	37	198	**0.106**	**0.106**	**0.106**	**0.106**	**0.106**	**0.106**	**0.106**	**0.106**	**0.106**
Chesapeake Lower	37	199	**0.141**	**0.142**	**0.142**	**0.142**	**0.142**	**0.142**	**0.142**	**0.142**	**0.142**
Chesapeake Middle	724	1,015	**0.219**	**0.219**	**0.219**	**0.219**	**0.219**	**0.219**	**0.219**	**0.219**	**0.219**
Chesapeake Upper	839	2,112	**0.201**	**0.201**	**0.201**	**0.201**	**0.201**	**0.201**	**0.201**	**0.201**	**0.201**
Codeminer	71	618	0.017	**0.018**	**0.018**	**0.018**	**0.018**	**0.018**	**0.018**	**0.018**	**0.018**
Cypress Dry	71	612	**0.134**	**0.134**	**0.134**	**0.134**	**0.134**	**0.134**	**0.134**	**0.134**	**0.134**
Cypress Wet	35	59	**0.129**	**0.129**	**0.129**	**0.129**	**0.129**	**0.129**	**0.129**	**0.129**	**0.129**
DNA Citation	39	61	**0.027**	**0.028**	**0.028**	**0.028**	**0.028**	**0.028**	**0.028**	**0.028**	**0.028**
E.Coli	418	519	0.005	**0.006**	**0.006**	**0.006**	**0.006**	**0.006**	**0.006**	**0.006**	**0.006**
Erdos 971	433	1,314	**0.081**	**0.081**	**0.081**	**0.081**	**0.081**	**0.081**	**0.081**	**0.081**	**0.081**
Erdos 981	445	1,381	**0.079**	**0.079**	**0.079**	**0.079**	**0.079**	**0.079**	**0.079**	**0.079**	**0.079**
Erdos 991	454	1,417	**0.085**	**0.085**	**0.085**	**0.085**	**0.085**	**0.085**	**0.085**	**0.085**	**0.085**
Everglades	69	880	**0.170**	**0.170**	**0.170**	**0.170**	**0.170**	**0.170**	**0.170**	**0.170**	**0.170**
Haggle Contact	1,226	2,408	**0.603**	**0.602**	**0.602**	**0.602**	**0.602**	**0.602**	**0.602**	**0.602**	**0.602**
Infectious	274	2,124	**0.278**	**0.278**	**0.278**	**0.278**	**0.278**	**0.278**	**0.278**	**0.278**	**0.278**
Japan Air	410	2,765	**0.181**	**0.182**	**0.182**	**0.182**	**0.182**	**0.182**	**0.182**	**0.182**	**0.182**
Jazz	56	183	**0.497**	**0.497**	**0.497**	**0.497**	**0.497**	**0.497**	**0.497**	**0.497**	**0.497**
Les Miserables	198	2,742	**0.521**	**0.521**	**0.521**	**0.521**	**0.521**	**0.521**	**0.521**	**0.521**	**0.521**
Macaque Neural	77	254	**0.545**	**0.545**	**0.545**	**0.545**	**0.545**	**0.545**	**0.545**	**0.545**	**0.545**
Manufacturing e-mail	94	1,515	**0.434**	**0.434**	**0.434**	**0.434**	**0.434**	**0.434**	**0.434**	**0.434**	**0.434**
Maspalomas	167	3,250	**0.123**	**0.123**	**0.123**	**0.123**	**0.123**	**0.123**	**0.123**	**0.123**	**0.123**
Physicians	24	77	0.065	**0.067**	**0.067**	**0.067**	**0.067**	**0.067**	**0.067**	**0.067**	**0.067**
Polbooks	35	204	**0.128**	**0.128**	**0.128**	**0.128**	**0.128**	**0.128**	**0.128**	**0.128**	**0.128**
Residence Hall	241	923	**0.175**	**0.175**	**0.175**	**0.175**	**0.175**	**0.175**	**0.175**	**0.175**	**0.175**
SFBD Food Web	105	441	**0.072**	**0.072**	**0.072**	**0.072**	**0.072**	**0.072**	**0.072**	**0.072**	**0.072**
SFBW Food Web	217	1,839	**0.072**	**0.072**	**0.072**	**0.072**	**0.072**	**0.072**	**0.072**	**0.072**	**0.072**
School	236	5,899	**0.371**	**0.371**	**0.371**	**0.371**	**0.371**	**0.371**	**0.371**	**0.371**	**0.371**
StMarks	128	2,106	**0.107**	**0.107**	**0.107**	**0.107**	**0.107**	**0.107**	**0.107**	**0.107**	**0.107**
Terrorist	128	2,075	**0.231**	**0.231**	**0.231**	**0.231**	**0.231**	**0.231**	**0.231**	**0.231**	**0.231**
Terrorist Train Bombing	54	350	**0.643**	**0.642**	**0.642**	**0.642**	**0.642**	**0.642**	**0.642**	**0.642**	**0.642**
US Air 97	62	152	**0.392**	**0.392**	**0.392**	**0.392**	**0.392**	**0.392**	**0.392**	**0.392**	**0.392**
World Trade	64	243	**0.324**	**0.324**	**0.324**	**0.324**	**0.324**	**0.324**	**0.324**	**0.324**	**0.324**
Zakarays Karate Club	332	2,126	**0.138**	**0.139**	**0.139**	**0.139**	**0.139**	**0.139**	**0.139**	**0.139**	**0.139**
Average significant rank			5.324	**4.959**	**4.959**	**4.959**	**4.959**	**4.959**	**4.959**	**4.959**	**4.959**

Table 5. Effect of the horizon cut-off h on the performance of Algorithm 1 in small networks (AUROC). We report the AUROC for different values of h averaged over 1000 test runs where 10% of the edges are removed while keeping the network connected. For every network, the results having the best significant rank with $p = 0.05$ are shown in bold. The last row shows the average significant rank over all networks (the lower the better). The columns n and m contain the number of nodes and edges in the network.

Network	n	m	$h=2$	$h=3$	$h=4$	$h=5$	$h=6$	$h=7$	$h=8$	$h=9$	$h=\infty$
ACM2009 Contacts	113	2,196	**0.784**	**0.784**	**0.784**	**0.784**	**0.784**	**0.784**	**0.784**	**0.784**	**0.784**
C.Elegans Metabolic	453	2,038	**0.948**	0.944	0.942	0.942	0.942	0.942	0.942	0.942	0.942
C.Elegans Neural	297	2,148	0.867	**0.872**	**0.872**	**0.872**	**0.872**	**0.872**	**0.872**	**0.872**	**0.872**
CPAN Authors	37	167	0.775	**0.807**	0.794	0.794	0.794	0.794	0.794	0.794	0.794
Centrality Literature	37	198	0.852	**0.855**	0.854	0.854	0.854	0.854	0.854	0.854	0.854
Chesapeake Lower	37	199	0.756	**0.769**	**0.769**	**0.769**	**0.769**	**0.769**	**0.769**	**0.769**	**0.769**
Chesapeake Middle	724	1,015	**0.805**	**0.806**	**0.806**	**0.806**	**0.806**	**0.806**	**0.806**	**0.806**	**0.806**
Chesapeake Upper	839	2,112	**0.789**	**0.790**	**0.790**	**0.790**	**0.790**	**0.790**	**0.790**	**0.790**	**0.790**
Codeminer	71	618	0.634	0.723	0.728	**0.740**	0.726	0.713	0.700	0.686	0.633
Cypress Dry	71	612	**0.796**	**0.798**	**0.798**	**0.798**	**0.798**	**0.798**	**0.798**	**0.798**	**0.798**
Cypress Wet	35	59	**0.791**	**0.793**	**0.793**	**0.793**	**0.793**	**0.793**	**0.793**	**0.793**	**0.793**
DNA Citation	39	61	0.606	0.633	**0.644**	0.640	0.639	0.638	0.638	0.638	0.638
E.Coli	418	519	0.562	**0.673**	0.644	0.629	0.609	0.599	0.592	0.589	0.587
Erdos 971	433	1,314	0.843	**0.865**	0.859	0.851	0.847	0.846	0.845	0.845	0.845
Erdos 981	445	1,381	0.846	**0.867**	0.861	0.853	0.851	0.850	0.849	0.849	0.849
Erdos 991	454	1,417	0.849	**0.870**	0.864	0.857	0.854	0.853	0.853	0.852	0.852
Everglades	69	880	**0.737**	**0.737**	**0.737**	**0.737**	**0.737**	**0.737**	**0.737**	**0.737**	**0.737**
Haggle Contact	1,226	2,408	**0.940**	0.932	0.931	0.931	0.931	0.931	0.931	0.931	0.931
Infectious	274	2,124	0.944	**0.959**	0.956	0.955	0.954	0.954	0.954	0.954	0.954
Japan Air	410	2,765	**0.837**	**0.841**	**0.840**	**0.840**	**0.840**	**0.840**	**0.840**	**0.840**	**0.840**
Jazz	56	183	**0.965**	0.965	0.965	0.965	0.965	0.965	0.965	0.965	0.965
Les Miserables	198	2,742	**0.911**	0.892	0.884	0.884	0.883	0.883	0.883	0.883	0.883
Macaque Neural	77	254	**0.949**	**0.948**	**0.948**	**0.948**	**0.948**	**0.948**	**0.948**	**0.948**	**0.948**
Manufacturing e-mail	94	1,515	**0.920**	0.920	0.920	0.920	0.920	0.920	0.920	0.920	0.920
Maspalomas	167	3,250	**0.690**	**0.688**	**0.687**	**0.687**	**0.687**	**0.687**	**0.687**	**0.687**	**0.687**
Physicians	24	77	0.843	0.916	**0.924**	**0.924**	**0.924**	**0.924**	**0.924**	**0.924**	**0.924**
Polbooks	35	204	0.895	**0.903**	0.899	0.897	0.897	0.897	0.897	0.897	0.897
Residence Hall	241	923	**0.897**	**0.897**	**0.896**	**0.896**	**0.896**	**0.896**	**0.896**	**0.896**	**0.896**
SFBD Food Web	105	441	**0.651**	**0.651**	**0.651**	**0.651**	**0.651**	**0.651**	**0.651**	**0.651**	**0.651**
SFBW Food Web	217	1,839	**0.655**	**0.655**	**0.655**	**0.655**	**0.655**	**0.655**	**0.655**	**0.655**	**0.655**
School	236	5,899	**0.909**	**0.909**	**0.909**	**0.909**	**0.909**	**0.909**	**0.909**	**0.909**	**0.909**
StMarks	128	2,106	**0.702**	**0.703**	**0.703**	**0.703**	**0.703**	**0.703**	**0.703**	**0.703**	**0.703**
Terrorist	128	2,075	**0.856**	**0.856**	0.846	0.842	0.841	0.841	0.841	0.841	0.841
Terrorist Train Bombing	54	350	**0.919**	0.915	0.910	0.908	0.908	0.908	0.908	0.908	0.908
US Air 97	62	152	**0.949**	0.942	0.939	0.938	0.938	0.938	0.938	0.938	0.938
World Trade	64	243	**0.837**	0.814	0.814	0.814	0.814	0.814	0.814	0.814	0.814
Zakarays Karate Club	332	2,126	0.745	**0.781**	**0.776**	**0.776**	**0.776**	**0.776**	**0.776**	**0.776**	**0.776**
Average significant rank			5.541	**3.770**	4.473	4.824	5.081	5.203	5.297	5.351	5.459

Table 6. Effect of the horizon cut-off h on the performance of Algorithm 1 in small networks (top-precision). We report the top-precision for different values of h averaged over 1000 test runs where 10% of the edges are removed while keeping the network connected. For every network, the results having the best significant rank with $p = 0.05$ are shown in bold. The last row shows the average significant rank over all networks (the lower the better). The columns n and m contain the number of nodes and edges in the network.

Network	n	m	$h=2$	$h=3$	$h=4$	$h=5$	$h=6$	$h=7$	$h=8$	$h=9$	$h=\infty$
ACM2009 Contacts	113	2,196	**0.273**	**0.273**	**0.273**	**0.273**	**0.273**	**0.273**	**0.273**	**0.273**	**0.273**
C.Elegans Metabolic	453	2,038	**0.187**	**0.187**	**0.187**	**0.187**	**0.187**	**0.187**	**0.187**	**0.187**	**0.187**
C.Elegans Neural	297	2,148	**0.108**	**0.108**	**0.108**	**0.108**	**0.108**	**0.108**	**0.108**	**0.108**	**0.108**
CPAN Authors	37	167	**0.105**	**0.105**	**0.105**	**0.105**	**0.105**	**0.105**	**0.105**	**0.105**	**0.105**
Centrality Literature	37	198	**0.176**	**0.176**	**0.176**	**0.176**	**0.176**	**0.176**	**0.176**	**0.176**	**0.176**
Chesapeake Lower	37	199	**0.197**	**0.197**	**0.197**	**0.197**	**0.197**	**0.197**	**0.197**	**0.197**	**0.197**
Chesapeake Middle	724	1,015	**0.258**	**0.258**	**0.258**	**0.258**	**0.258**	**0.258**	**0.258**	**0.258**	**0.258**
Chesapeake Upper	839	2,112	**0.291**	**0.291**	**0.291**	**0.291**	**0.291**	**0.291**	**0.291**	**0.291**	**0.291**
Codeminer	71	618	**0.057**	**0.057**	**0.057**	**0.057**	**0.057**	**0.057**	**0.057**	**0.057**	**0.057**
Cypress Dry	71	612	**0.202**	**0.202**	**0.202**	**0.202**	**0.202**	**0.202**	**0.202**	**0.202**	**0.202**
Cypress Wet	35	59	**0.213**	**0.213**	**0.213**	**0.213**	**0.213**	**0.213**	**0.213**	**0.213**	**0.213**
DNA Citation	39	61	**0.047**	**0.047**	**0.047**	**0.047**	**0.047**	**0.047**	**0.047**	**0.047**	**0.047**
E.Coli	418	519	**0.034**	**0.034**	**0.034**	**0.034**	**0.034**	**0.034**	**0.034**	**0.034**	**0.034**
Erdos 971	433	1,314	**0.152**	**0.152**	**0.152**	**0.152**	**0.152**	**0.152**	**0.152**	**0.152**	**0.152**
Erdos 981	445	1,381	**0.151**	**0.151**	**0.151**	**0.151**	**0.151**	**0.151**	**0.151**	**0.151**	**0.151**
Erdos 991	454	1,417	**0.154**	**0.154**	**0.154**	**0.154**	**0.154**	**0.154**	**0.154**	**0.154**	**0.154**
Everglades	69	880	**0.243**	**0.243**	**0.243**	**0.243**	**0.243**	**0.243**	**0.243**	**0.243**	**0.243**
Haggle Contact	1,226	2,408	**0.566**	**0.566**	**0.566**	**0.566**	**0.566**	**0.566**	**0.566**	**0.566**	**0.566**
Infectious	274	2,124	**0.344**	**0.344**	**0.344**	**0.344**	**0.344**	**0.344**	**0.344**	**0.344**	**0.344**
Japan Air	410	2,765	**0.223**	**0.223**	**0.223**	**0.223**	**0.223**	**0.223**	**0.223**	**0.223**	**0.223**
Jazz	56	183	**0.521**	**0.521**	**0.521**	**0.521**	**0.521**	**0.521**	**0.521**	**0.521**	**0.521**
Les Miserables	198	2,742	**0.547**	**0.547**	**0.547**	**0.547**	**0.547**	**0.547**	**0.547**	**0.547**	**0.547**
Macaque Neural	77	254	**0.559**	**0.559**	**0.559**	**0.559**	**0.559**	**0.559**	**0.559**	**0.559**	**0.559**
Manufacturing e-mail	94	1,515	**0.424**	**0.424**	**0.424**	**0.424**	**0.424**	**0.424**	**0.424**	**0.424**	**0.424**
Maspalomas	167	3,250	**0.172**	**0.172**	**0.172**	**0.172**	**0.172**	**0.172**	**0.172**	**0.172**	**0.172**
Physicians	24	77	**0.126**	**0.126**	**0.126**	**0.126**	**0.126**	**0.126**	**0.126**	**0.126**	**0.126**
Polbooks	35	204	**0.200**	**0.200**	**0.200**	**0.200**	**0.200**	**0.200**	**0.200**	**0.200**	**0.200**
Residence Hall	241	923	**0.246**	**0.246**	**0.246**	**0.246**	**0.246**	**0.246**	**0.246**	**0.246**	**0.246**
SFBD Food Web	105	441	**0.121**	**0.121**	**0.121**	**0.121**	**0.121**	**0.121**	**0.121**	**0.121**	**0.121**
SFBW Food Web	217	1,839	**0.119**	**0.119**	**0.119**	**0.119**	**0.119**	**0.119**	**0.119**	**0.119**	**0.119**
School	236	5,899	**0.409**	**0.409**	**0.409**	**0.409**	**0.409**	**0.409**	**0.409**	**0.409**	**0.409**
StMarks	128	2,106	**0.183**	**0.183**	**0.183**	**0.183**	**0.183**	**0.183**	**0.183**	**0.183**	**0.183**
Terrorist	128	2,075	**0.294**	**0.294**	**0.294**	**0.294**	**0.294**	**0.294**	**0.294**	**0.294**	**0.294**
Terrorist Train Bombing	54	350	**0.703**	**0.703**	**0.703**	**0.703**	**0.703**	**0.703**	**0.703**	**0.703**	**0.703**
US Air 97	62	152	**0.448**	**0.448**	**0.448**	**0.448**	**0.448**	**0.448**	**0.448**	**0.448**	**0.448**
World Trade	64	243	**0.400**	**0.400**	**0.400**	**0.400**	**0.400**	**0.400**	**0.400**	**0.400**	**0.400**
Zakarays Karate Club	332	2,126	**0.153**	**0.153**	**0.153**	**0.153**	**0.153**	**0.153**	**0.153**	**0.153**	**0.153**
Average significant rank			**5.000**	**5.000**	**5.000**	**5.000**	**5.000**	**5.000**	**5.000**	**5.000**	**5.000**

Table 7. Effect of the horizon cut-off h on the performance of Algorithm 1 in large networks. We report top-precision for different values of h averaged over 100 test runs where 10% of the edges are removed while keeping the network connected. For every network, the results having the best significant rank with $p = 0.05$ are shown in bold. The last row shows the average significant rank over all networks (the lower the better). The columns n and m contain the number of nodes and edges in the network.

Network	n	m	$h=2$	$h=3$	$h=4$	$h=5$	$h=6$	$h=7$	$h=8$	$h=9$	$h=\infty$
AS CAIDA 2007 11 05	5,155	39,285	**0.053**	**0.053**	**0.053**	**0.053**	**0.053**	**0.053**	**0.053**	**0.053**	**0.053**
AS Internet	26,475	53,381	**0.115**	**0.115**	**0.115**	**0.115**	**0.115**	**0.115**	**0.115**	**0.115**	**0.115**
Advogato	17,258	45,702	**0.158**	**0.158**	**0.158**	**0.158**	**0.158**	**0.158**	**0.158**	**0.158**	**0.158**
Astro	18,771	198,050	**0.500**	**0.500**	**0.500**	**0.500**	**0.500**	**0.500**	**0.500**	**0.500**	**0.500**
Bitcoin	5,881	21,492	**0.104**	**0.104**	**0.104**	**0.104**	**0.104**	**0.104**	**0.104**	**0.104**	**0.104**
Cond Mat	23,133	93,439	**0.521**	**0.521**	**0.521**	**0.521**	**0.521**	**0.521**	**0.521**	**0.521**	**0.521**
Criminal	2,749	2,952	**0.053**	**0.053**	**0.053**	**0.053**	**0.053**	**0.053**	**0.053**	**0.053**	**0.053**
Diseasome	1,419	2,738	**0.573**	**0.573**	**0.573**	**0.573**	**0.573**	**0.573**	**0.573**	**0.573**	**0.573**
EAT	23,219	304,938	**0.049**	**0.049**	**0.049**	**0.049**	**0.049**	**0.049**	**0.049**	**0.049**	**0.049**
EVA	1,133	5,451	**0.009**	**0.009**	**0.009**	**0.009**	**0.009**	**0.009**	**0.009**	**0.009**	**0.009**
Email	6,927	11,850	**0.161**	**0.161**	**0.161**	**0.161**	**0.161**	**0.161**	**0.161**	**0.161**	**0.161**
Erdos 02	7,253	6,711	**0.081**	**0.081**	**0.081**	**0.081**	**0.081**	**0.081**	**0.081**	**0.081**	**0.081**
GR	6,301	20,777	**0.522**	**0.522**	**0.522**	**0.522**	**0.522**	**0.522**	**0.522**	**0.522**	**0.522**
G Plus	22,687	54,705	**0.035**	**0.035**	**0.035**	**0.035**	**0.035**	**0.035**	**0.035**	**0.035**	**0.035**
Gnutella 08	23,628	39,194	**0.013**	**0.014**	**0.014**	**0.014**	**0.014**	**0.014**	**0.014**	**0.014**	**0.014**
Gnutella 25	5,241	14,484	**0.004**	0.004	0.004	0.004	0.004	0.004	0.004	0.004	0.004
HepTh Cit	27,769	352,285	**0.152**	**0.152**	**0.152**	**0.152**	**0.152**	**0.152**	**0.152**	**0.152**	**0.152**
Hero	10,469	178,115	**0.340**	**0.340**	**0.340**	**0.340**	**0.340**	**0.340**	**0.340**	**0.340**	**0.340**
Indochina 2004	11,358	47,606	**0.763**	**0.763**	**0.763**	**0.763**	**0.763**	**0.763**	**0.763**	**0.763**	**0.763**
Java	1,538	7,817	**0.131**	**0.131**	**0.131**	**0.131**	**0.131**	**0.131**	**0.131**	**0.131**	**0.131**
Lederberg	8,324	41,532	**0.129**	**0.129**	**0.129**	**0.129**	**0.129**	**0.129**	**0.129**	**0.129**	**0.129**
Mathoverflow C2A	13,778	71,234	**0.119**	**0.119**	**0.119**	**0.119**	**0.119**	**0.119**	**0.119**	**0.119**	**0.119**
Net Science	1,461	2,742	**0.671**	**0.671**	**0.671**	**0.671**	**0.671**	**0.671**	**0.671**	**0.670**	**0.671**
ODLIS	1,899	13,838	**0.118**	**0.118**	**0.118**	**0.118**	**0.118**	**0.118**	**0.118**	**0.118**	**0.118**
Oclinks	2,900	16,377	**0.046**	**0.046**	**0.046**	**0.046**	**0.046**	**0.046**	**0.046**	**0.046**	**0.046**
Oregon	11,174	23,409	**0.073**	**0.073**	**0.073**	**0.073**	**0.073**	**0.073**	**0.073**	**0.073**	**0.073**
PGP	10,680	24,316	**0.344**	**0.344**	**0.344**	**0.344**	**0.344**	**0.344**	**0.344**	**0.344**	**0.344**
Political Blogs	643	2,280	**0.160**	**0.160**	**0.160**	**0.160**	**0.160**	**0.160**	**0.160**	**0.160**	**0.160**
Power	4,941	6,594	**0.039**	**0.039**	**0.039**	**0.039**	**0.039**	**0.039**	**0.039**	**0.039**	**0.039**
Reuters 9 11	13,314	148,038	**0.139**	**0.139**	**0.139**	**0.139**	**0.139**	**0.139**	**0.139**	**0.139**	**0.139**
Roget	1,010	3,648	**0.067**	**0.067**	**0.067**	**0.067**	**0.067**	**0.067**	**0.067**	**0.067**	**0.067**
Spam	4,767	37,375	**0.175**	**0.175**	**0.175**	**0.175**	**0.175**	**0.175**	**0.175**	**0.175**	**0.175**
Terror	4,275	6,531	**0.140**	**0.140**	**0.140**	**0.140**	**0.140**	**0.140**	**0.140**	**0.140**	**0.140**
Web-Base 2001	16,062	25,593	**0.175**	**0.175**	**0.175**	**0.175**	**0.175**	**0.175**	**0.175**	**0.175**	**0.175**
Web EPA	3,031	6,474	**0.028**	**0.028**	**0.028**	**0.028**	**0.028**	**0.028**	**0.028**	**0.028**	**0.028**
Web Edu	4,271	8,909	**0.414**	**0.414**	**0.414**	**0.414**	**0.414**	**0.414**	**0.414**	**0.414**	**0.414**
WikiVote	7,115	100,762	**0.107**	**0.107**	**0.107**	**0.107**	**0.107**	**0.107**	**0.107**	**0.107**	**0.107**
World Air	3,618	14,142	**0.249**	**0.249**	**0.249**	**0.249**	**0.249**	**0.249**	**0.249**	**0.249**	**0.249**
Yeast	2,284	6,646	**0.110**	**0.110**	**0.110**	**0.110**	**0.110**	**0.110**	**0.110**	**0.110**	**0.110**
Youtube	13,723	76,764	**0.097**	**0.097**	**0.097**	**0.097**	**0.097**	**0.097**	**0.097**	**0.097**	**0.097**
Average significant rank			**4.900**	4.925	5.025	5.025	5.025	5.025	5.025	5.025	5.025

Table 8. Comparison of Algorithm 1 with global link prediction methods on small networks. We report top-precision averaged over 100 test runs where 10% of the edges are removed while keeping the network connected. For every network, the results having the best significant rank with $p = 0.05$ are shown in bold. The last row shows the average significant rank over all networks (the lower the better). The columns n and m contain the number of nodes and edges in the network.

Network	n	m	FBM	HRG	HYP	SBM	ALG1
ACM2009 Contacts	113	2,196	0.2600	0.2320	0.0884	0.2401	**0.2801**
C.Elegans Metabolic	453	2,038	0.1666	0.1137	0.0999	**0.2091**	**0.2111**
C.Elegans Neural	297	2,148	0.1180	0.0746	0.0468	**0.1489**	0.1150
DNA Citation CC	35	59	0.0333	**0.0717**	0.0450	0.0500	0.0617
Haggle Contact	274	2,124	0.5895	0.3928	0.1766	**0.6451**	0.6223
Infectious	410	2,765	0.3436	0.2670	0.1276	0.3058	**0.3636**
Japan Air	56	183	**0.2589**	0.1767	0.0389	**0.2644**	**0.2697**
Jazz	198	2,742	0.4585	0.3159	0.2278	0.4770	**0.5304**
Les Miserables	77	254	0.5348	0.3172	0.1752	0.5643	**0.5982**
Macaque Neural	94	1,515	0.5603	0.4314	0.2936	**0.6751**	0.5643
Manufacturing e-mail	167	3,250	0.3915	0.4208	0.1374	**0.4877**	0.4349
Narragan	35	204	0.2480	**0.4760**	0.1090	**0.4725**	0.3480
Polbooks	105	441	0.1754	0.1064	0.0771	0.1615	**0.2220**
Political Blogs	643	2,280	0.1744	0.0946	0.0103	0.1471	**0.2013**
Residence Hall	217	1,839	**0.2513**	0.1538	0.1665	0.1898	**0.2509**
Terrorist	62	152	0.2773	0.2713	0.0707	0.3120	**0.3680**
Terrorist Train Bombing	64	243	0.5538	0.5137	0.2621	**0.7287**	**0.7454**
US Air 97	332	2,126	0.4147	0.2269	0.2501	0.4142	**0.4910**
Average significant rank			2.8056	3.6944	4.8611	2.0556	**1.5833**

Table 9. Comparison of Algorithm 1 with local link prediction methods on small networks. We report top-precision averaged over 1000 test runs where 10% of the edges are removed at each run and used as test set. For every network, the results having the best significant rank with $p = 0.05$ are shown in bold. The last row shows the average significant rank over all networks (the lower the better). The columns n and m contain the number of nodes and edges in the network.

Network	n	m	ADA	CNE	CH	HPI	JID	PAT	RAL	ALG1
ACM2009 Contacts	113	2,196	0.2724	0.2709	0.2708	0.1002	0.2447	0.2661	**0.2734**	0.2731
C.Elegans Metabolic	453	2,038	0.1945	0.1401	0.2022	0.1069	0.0485	0.1034	**0.2687**	0.1880
C.Elegans Neural	297	2,148	0.1056	0.0956	**0.1150**	0.0097	0.0234	0.0573	0.1026	0.1076
Centrality Literature	118	613	0.1320	0.1161	0.1411	0.0395	0.0354	0.1595	0.1437	**0.1736**
Chesapeake Lower	37	167	0.1506	0.1303	0.1345	0.0357	0.0106	**0.2299**	0.1684	0.1965
Chesapeake Middle	37	198	0.2318	0.2238	0.2438	0.1068	0.0521	0.2308	0.2382	**0.2593**
Chesapeake Upper	37	199	0.2440	0.2183	0.1978	0.0606	0.0360	**0.3329**	0.2612	0.2930
Codeminer	724	1,015	0.0429	0.0266	0.0137	0.0230	0.0005	0.0033	0.0478	**0.0557**
CPAN Authors	839	2,112	0.1021	0.0918	0.0925	0.0023	0.0000	0.0882	0.0946	**0.1058**
Cypress Dry	71	618	0.1501	0.1425	0.1560	0.0095	0.0255	**0.2408**	0.1599	0.2018
Cypress Wet	71	612	0.1554	0.1442	0.1531	0.0125	0.0205	**0.2368**	0.1681	0.2163
DNA Citation CC	35	59	0.0260	0.0197	0.0085	0.0250	0.0162	0.0255	0.0275	**0.0465**
DNA Citation	39	61	0.0332	0.0232	0.0107	0.0253	0.0198	0.0245	0.0332	**0.0470**
E.Coli	418	519	0.0243	0.0088	0.0009	0.0012	0.0003	0.0118	0.0278	**0.0334**
Erdos 971	433	1,314	**0.1526**	0.1244	0.1398	0.0352	0.0515	0.0662	**0.1516**	**0.1534**
Erdos 981	445	1,381	**0.1527**	0.1252	0.1398	0.0362	0.0514	0.0668	**0.1530**	**0.1515**
Erdos 991	454	1,417	0.1543	0.1244	0.1434	0.0393	0.0531	0.0646	**0.1609**	0.1546
Everglades	69	880	0.1636	0.1550	0.1583	0.0521	0.0184	**0.3160**	0.1756	0.2406
GD 01	259	640	**0.1209**	0.1115	0.1105	0.0243	0.0571	0.0124	0.1014	**0.1209**
Haggle Contact	274	2,124	0.5526	0.5511	0.5520	0.0140	0.0000	**0.6035**	0.5529	0.5677
Infectious	410	2,765	0.3282	0.3057	0.3340	0.1114	**0.3457**	0.0245	0.3443	0.3432
Japan Air	56	183	0.1924	0.1746	0.1808	0.0236	0.0000	**0.2675**	0.1965	0.2216
Jazz	198	2,742	0.5216	0.5026	**0.5589**	0.2489	0.5166	0.1306	0.5393	0.5221
Les Miserables	77	254	0.5255	0.4739	0.5156	0.2165	0.0658	0.1002	0.5413	**0.5487**
Macaque Neural	94	1,515	0.5286	0.5331	**0.5575**	0.0275	0.3678	0.2752	0.5125	**0.5575**
Manufacturing e-mail	167	3,250	0.4137	0.4085	0.4156	0.0173	0.2657	0.3717	**0.4228**	**0.4238**
Maspalomas	24	77	0.1212	0.0931	0.0885	0.0499	0.0200	**0.1665**	0.1365	**0.1751**
Narragan	35	204	0.2713	0.2392	0.2362	0.0936	0.0380	0.3192	0.2957	**0.3306**
Physicians	241	923	0.1236	0.1144	0.1199	0.0894	0.0933	0.0121	0.1207	**0.1267**
Polbooks	105	441	0.1951	0.1692	0.1700	0.1256	0.1091	0.0447	**0.2004**	**0.2004**
Political Blogs	643	2,280	0.1519	0.1420	0.1452	0.0079	0.0002	0.0298	0.1376	**0.1595**
Residence Hall	217	1,839	0.2389	0.2152	0.2485	0.2220	**0.2703**	0.0506	0.2557	0.2435
School	236	5,899	0.2906	0.2728	0.3796	0.3960	0.4012	0.1138	0.3662	**0.4090**
SFBD Food Web	128	2,106	0.0724	0.0700	0.0860	0.0566	0.0113	**0.1654**	0.0725	0.1215
SFBW Food Web	128	2,075	0.0739	0.0716	0.0842	0.0583	0.0107	**0.1642**	0.0738	0.1197
StMarks	54	350	0.1517	0.1371	0.1263	0.0611	0.0342	**0.2346**	0.1571	0.1844
Terrorist	62	152	**0.2923**	0.2443	0.2762	0.1568	0.0549	0.1059	**0.2907**	**0.2936**
Terrorist Train Bombing	64	243	0.6798	0.5720	0.6696	0.1908	0.4971	0.2245	**0.7075**	**0.7069**
US Air 97	332	2,126	0.3921	0.3722	0.3914	0.0376	0.0730	0.3212	**0.4555**	0.4496
Zakarays Karate Club	34	78	0.1345	0.1305	**0.1921**	0.0216	0.0016	0.0709	0.1430	0.1554
Average significant rank			3.5125	5.2250	4.1250	6.8250	6.9125	4.7750	2.8250	**1.8000**

Table 10. Comparison of Algorithm 1 with local link prediction methods on large networks. We report top-precision averaged over 100 test runs where 10% of the edges are removed at each run and used as test set. For every network, the results having the best significant rank with $p = 0.05$ are shown in bold. The last row shows the average significant rank over all networks (the lower the better).The columns n and m contain the number of nodes and edges in the network.

Network	n	m	ADA	CNE	CH	HPI	JID	PAT	RAL	ALG1
Advogato	5,155	39,285	0.1329	0.1214	**0.1548**	0.0122	0.0023	0.0502	0.1390	**0.1539**
AS CAIDA 2007 11 05	26,475	53,381	0.0480	0.0444	0.0488	0.0004	0.0000	0.0327	0.0350	**0.0534**
AS Internet	17,258	45,702	**0.1124**	0.0861	0.1108	0.0022	0.0001	0.0664	0.1107	**0.1135**
Astro	18,771	198,050	0.4499	0.3824	0.5052	0.0948	0.3409	0.0203	**0.6307**	0.5640
Bitcoin	5,881	21,492	0.0953	0.0884	**0.1099**	0.0047	0.0004	0.0504	0.0859	0.1057
Cond Mat	23,133	93,439	0.4847	0.3318	0.4866	0.2491	0.2404	0.0090	**0.5485**	0.5074
Criminal	2,749	2,952	0.0273	0.0416	**0.0452**	0.0033	0.0000	0.0232	0.0219	0.0413
Diseasome	1,419	2,738	**0.5891**	0.3822	0.5046	0.3384	0.0689	0.0152	**0.5935**	0.4906
EAT	23,219	304,938	0.0665	0.0652	**0.0911**	0.0016	0.0003	0.0126	0.0413	0.0651
Email	1,133	5,451	0.1552	0.1407	0.1576	0.0137	0.0696	0.0170	0.1417	**0.1624**
Erdos 02	6,927	11,850	0.0772	0.0754	0.0727	0.0070	0.0000	0.0455	0.0635	**0.0819**
EVA	7,253	6,711	0.0068	0.0035	0.0006	0.0007	0.0000	0.0012	0.0067	**0.0080**
FAA	1,226	2,408	0.0207	**0.0294**	0.0219	0.0062	0.0008	0.0070	0.0155	**0.0283**
Gnutella 08	6,301	20,777	0.0096	0.0101	0.0081	0.0002	0.0000	**0.0109**	0.0087	0.0100
Gnutella 25	22,687	54,705	0.0014	**0.0019**	0.0008	0.0002	0.0000	0.0006	0.0014	**0.0020**
G Plus	23,628	39,194	0.0187	0.0162	0.0219	0.0003	0.0000	0.0112	0.0164	**0.0323**
GR	5,241	14,484	0.4860	0.3627	0.4671	0.1971	0.3562	0.0582	**0.5490**	0.4964
HepTh Cit	27,769	352,285	0.1849	0.1698	**0.2066**	0.0094	0.1210	0.0194	0.1976	0.1584
Hero	10,469	178,115	0.2426	0.2163	0.3394	0.0066	0.0019	0.1239	**0.4427**	0.3788
Indochina 2004	11,358	47,606	0.8226	0.7528	0.8114	0.2763	0.0844	0.0142	0.8300	**0.8404**
Java	1,538	7,817	0.1203	0.1079	0.1259	0.0142	0.0002	0.1029	0.1185	**0.1401**
Lederberg	8,324	41,532	0.1183	0.0996	0.1227	0.0168	0.0008	0.0131	0.1208	**0.1258**
Mathoverflow C2A	13,778	71,234	**0.1239**	0.1192	**0.1234**	0.0014	0.0000	0.0930	0.1172	0.1199
Net Science	1,461	2,742	0.6708	0.4585	0.5782	0.4501	0.5230	0.0355	**0.6858**	0.5917
Oclinks	1,899	13,838	0.0255	0.0239	**0.0459**	0.0013	0.0000	0.0437	0.0275	**0.0464**
ODLIS	2,900	16,377	0.1030	0.0936	**0.1198**	0.0250	0.0156	0.0352	0.1017	0.1105
Oregon	11,174	23,409	0.0673	0.0621	0.0682	0.0007	0.0000	0.0447	0.0471	**0.0752**
PGP	10,680	24,316	0.3110	0.2591	0.3136	0.0824	0.0107	0.0212	0.2831	**0.3434**
Power	4,941	6,594	0.0272	**0.0423**	0.0345	0.0206	0.0102	0.0004	0.0247	0.0362
Reuters 9 11	13,314	148,038	0.1193	0.1156	0.1196	0.0068	0.0057	0.1056	**0.1425**	0.1386
Roget	1,010	3,648	**0.0807**	**0.0833**	0.0629	0.0256	0.0508	0.0070	0.0675	0.0690
Spam	4,767	37,375	0.1289	0.1081	0.1538	0.0183	0.0335	0.0680	0.1664	**0.1728**
Terror	4,275	6,531	**0.1571**	0.1144	0.1224	0.0302	0.0029	0.0053	0.1417	0.1426
Web-Base 2001	16,062	25,593	0.1734	0.1591	0.1690	0.0047	0.0007	0.0031	0.1630	**0.1756**
Web Edu	3,031	6,474	0.4267	0.2273	0.1928	0.2675	0.0103	0.0121	0.4247	**0.5032**
Web EPA	4,271	8,909	0.0150	0.0087	0.0134	0.0021	0.0001	0.0043	0.0200	**0.0270**
WikiVote	7,115	100,762	0.0992	0.1000	**0.1126**	0.0009	0.0001	0.0607	0.0831	0.1063
World Air	3,618	14,142	0.2587	0.2286	0.2532	0.0232	0.0091	0.0884	0.2883	**0.3001**
Yeast	2,284	6,646	0.0910	0.0882	0.1058	0.0177	0.0003	0.0094	0.0747	**0.1096**
Youtube	13,723	76,764	0.0867	0.0777	0.0942	0.0072	0.0018	0.0109	0.0790	**0.1025**
Average significant rank			3.0375	4.1750	2.9250	6.7500	7.5500	6.3750	3.3500	**1.8375**

Table 11. Comparison of Algorithm 1 with local link prediction methods on very large networks. We report top-precision averaged over 100 test runs where 10% of the edges are removed at each run and used as test set. For every network, the results having the best significant rank with $p = 0.05$ are shown in bold. The last row shows the average significant rank over all networks (the lower the better).The columns n and m contain the number of nodes and edges in the network.

Network	n	m	ADA	CNE	CH	HPI	JID	PAT	RAL	ALG1
Amazon 03 02	262,111	899,792	**0.2351**	0.1911	0.2178	0.1896	0.2119	0.0006	0.2328	0.2275
Amazon	334,863	925,872	0.1677	0.1272	0.1636	0.0888	0.0345	0.0004	0.1584	**0.1850**
Askubuntu A2Q	134,035	246,781	0.0245	0.0255	0.0248	0.0003	0.0000	0.0180	0.0085	**0.0261**
Brightkite	58,228	214,078	0.1155	0.0963	0.1204	0.0162	0.0016	0.0189	0.1404	**0.1554**
Deezer HR	54,573	498,202	0.1145	0.1035	0.1226	0.0119	0.0845	0.0034	0.1110	**0.1257**
Deezer HU	47,538	222,887	0.0915	0.0885	0.0919	0.0076	0.0508	0.0017	0.0626	**0.0982**
Deezer RO	41,773	125,826	0.0466	0.0520	0.0572	0.0087	0.0221	0.0015	0.0446	**0.0638**
Digg Reply	30,360	85,155	0.0043	0.0069	0.0028	0.0000	0.0000	**0.0097**	0.0014	0.0043
Douban	154,908	327,162	0.0141	0.0180	0.0174	0.0012	0.0000	0.0032	0.0081	**0.0198**
Enron Email	36,692	183,831	0.1564	0.1326	0.2130	0.0346	0.0177	0.0318	**0.3271**	0.2561
Epinions	75,879	405,740	0.0831	0.0764	0.1067	0.0064	0.0004	0.0384	0.0914	**0.1079**
Facebook 2	50,515	819,090	0.1756	0.1569	0.1929	0.0059	0.1308	0.0108	**0.2168**	0.1893
Facebook Artists	63,392	816,886	0.0260	0.0216	0.0674	0.0019	0.0187	0.0156	0.0448	**0.0862**
Func-Func	46,027	106,510	0.0546	0.0395	0.0007	0.0060	0.0000	0.0497	0.0437	**0.0799**
Gnutella 31	62,586	147,892	0.0019	0.0016	0.0007	0.0001	0.0000	0.0003	0.0018	**0.0023**
Gowalla	196,591	950,327	0.1052	0.0794	0.1394	0.0071	0.0009	0.0204	0.1410	**0.1454**
HepPh Cit	34,546	420,877	0.1541	0.1391	**0.1737**	0.0081	0.0865	0.0056	0.1628	0.1591
Internet	124,651	193,620	0.0140	0.0079	0.0148	0.0030	0.0013	0.0013	0.0142	**0.0249**
Livemocha	104,103	2,193,083	0.0119	0.0116	0.0262	0.0002	0.0000	0.0152	0.0113	**0.0307**
SK 2005	121,422	334,419	0.5524	0.4329	0.5690	0.0582	0.0194	0.0013	0.4584	**0.5701**
Slashdot 09 02	82,168	504,230	0.0409	0.0401	0.0461	0.0004	0.0002	0.0164	0.0280	**0.0483**
Superuser C2A	100,391	263,973	**0.0591**	0.0570	0.0575	0.0006	0.0000	0.0462	0.0368	0.0551
Twitter Follows	404,719	713,319	0.0017	0.0008	0.0017	0.0003	0.0000	0.0013	0.0021	**0.0025**
US Patents	240,547	560,943	0.0202	0.0178	0.0233	0.0035	0.0006	0.0009	0.0129	**0.0309**
Wordnet	73,753	234,024	0.1798	0.1166	0.2146	0.0166	0.0071	0.0067	0.1974	**0.2231**
Yahoo IM	100,001	587,964	0.1125	0.0818	0.1644	0.0294	0.0536	0.0020	0.1316	**0.1701**
Average significant rank			3.3077	4.4231	2.7692	6.8077	7.2115	6.3654	3.6346	**1.4808**

Table 12. Comparison of Algorithm 1 with local link prediction methods on small networks. We report top-precision averaged over 1000 test runs where 10% of the edges are removed at each run and used as test set. For every network, the results having the best significant rank with $p = 0.05$ are shown in bold. The last row shows the average significant rank over all networks (the lower the better). The columns n and m contain the number of nodes and edges in the network.

Network	ADA	CNE	CH	HDI	HPI	JID	LHN	PAT	RAL	RND	SAI	SHP	SOI	ALG1
ACM2009 Contacts	0.272	0.271	0.271	0.212	0.100	0.245	0.022	0.266	**0.273**	0.050	0.251	0.051	0.245	0.273
C.Elegans Metabolic	0.194	0.140	0.202	0.036	0.107	0.049	0.042	0.103	**0.269**	0.002	0.052	0.005	0.049	0.188
C.Elegans Neural	0.106	0.096	**0.115**	0.024	0.010	0.023	0.000	0.057	0.103	0.005	0.022	0.010	0.023	0.108
Centrality Literature	0.132	0.116	0.141	0.035	0.040	0.035	0.003	0.160	0.144	0.010	0.036	0.017	0.035	**0.174**
Chesapeake Lower	0.151	0.130	0.135	0.013	0.036	0.011	0.007	**0.230**	0.168	0.032	0.011	0.030	0.011	0.197
Chesapeake Middle	0.232	0.224	0.244	0.052	0.107	0.052	0.011	0.231	0.238	0.041	0.052	0.043	0.052	**0.259**
Chesapeake Upper	0.244	0.218	0.198	0.042	0.061	0.036	0.014	**0.333**	0.261	0.040	0.035	0.041	0.036	0.293
Codeminer	0.043	0.027	0.014	0.001	0.023	0.000	0.001	0.003	0.048	0.000	0.000	0.005	0.001	**0.056**
CPAN Authors	0.102	0.092	0.093	0.000	0.002	0.000	0.000	0.088	0.095	0.001	0.000	0.001	0.000	**0.106**
Cypress Dry	0.150	0.142	0.156	0.027	0.010	0.025	0.000	**0.241**	0.160	0.031	0.025	0.034	0.025	0.202
Cypress Wet	0.155	0.144	0.153	0.021	0.013	0.020	0.000	**0.237**	0.168	0.030	0.020	0.034	0.020	0.216
DNA Citation CC	0.026	0.020	0.009	0.019	0.025	0.016	0.019	0.026	0.028	0.012	0.017	0.019	0.016	**0.047**
DNA Citation	0.033	0.023	0.011	0.021	0.025	0.020	0.023	0.025	0.033	0.010	0.020	0.017	0.020	**0.047**
E.Coli	0.024	0.009	0.001	0.000	0.001	0.000	0.001	0.012	0.028	0.001	0.000	0.002	0.001	**0.033**
Erdos 971	**0.153**	0.124	0.140	0.052	0.035	0.051	0.018	0.066	**0.152**	0.001	0.042	0.014	0.051	**0.153**
Erdos 981	**0.153**	0.125	0.140	0.056	0.036	0.051	0.018	0.067	**0.153**	0.001	0.045	0.013	0.051	**0.151**
Erdos 991	0.154	0.124	0.143	0.057	0.039	0.053	0.020	0.065	**0.161**	0.001	0.047	0.013	0.053	0.155
Everglades	0.164	0.155	0.158	0.017	0.052	0.018	0.011	**0.316**	0.176	0.058	0.019	0.058	0.018	0.241
GD 01	**0.121**	0.112	0.110	0.060	0.024	0.057	0.021	0.012	0.101	0.002	0.053	0.017	0.057	**0.121**
Haggle Contact	0.553	0.551	0.552	0.000	0.014	0.000	0.000	**0.604**	0.553	0.006	0.000	0.013	0.000	0.568
Infectious	0.328	0.306	0.334	0.331	0.111	**0.346**	0.071	0.024	0.344	0.003	0.344	0.022	**0.346**	0.343
Japan Air	0.192	0.175	0.181	0.000	0.024	0.000	0.000	**0.267**	0.196	0.013	0.000	0.020	0.000	0.222
Jazz	0.522	0.503	**0.559**	0.466	0.249	0.517	0.099	0.131	0.539	0.015	0.529	0.026	0.517	0.522
Les Miserables	0.526	0.474	0.516	0.067	0.216	0.066	0.003	0.100	0.541	0.009	0.065	0.024	0.063	**0.549**
Macaque Neural	0.529	0.533	**0.558**	0.371	0.027	0.368	0.014	0.275	0.513	0.050	0.366	0.062	0.368	**0.557**
Manufacturing e-mail	0.414	0.408	0.416	0.224	0.017	0.266	0.000	0.372	**0.423**	0.030	0.283	0.040	0.266	**0.424**
Maspalomas	0.121	0.093	0.088	0.017	0.050	0.020	0.011	**0.167**	0.137	0.039	0.022	0.041	0.020	**0.175**
Narragan	0.271	0.239	0.236	0.043	0.094	0.038	0.019	0.319	0.296	0.051	0.041	0.049	0.038	**0.331**
Physicians	0.124	0.114	0.120	0.085	0.089	0.093	0.048	0.012	0.121	0.003	0.097	0.022	0.093	**0.127**
Polbooks	0.195	0.169	0.170	0.100	0.126	0.109	0.070	0.045	**0.200**	0.008	0.111	0.029	0.109	**0.200**
Political Blogs	0.152	0.142	0.145	0.000	0.008	0.000	0.000	0.030	0.138	0.001	0.000	0.006	0.000	**0.160**
Residence Hall	0.239	0.215	0.249	0.254	0.222	**0.270**	0.169	0.051	0.256	0.009	**0.272**	0.018	**0.270**	0.244
School	0.291	0.273	0.380	0.353	0.396	0.401	0.332	0.114	0.366	0.026	**0.416**	0.030	0.401	0.409
SFBD Food Web	0.072	0.070	0.086	0.010	0.057	0.011	0.014	**0.165**	0.073	0.033	0.012	0.036	0.011	0.121
SFBW Food Web	0.074	0.072	0.084	0.010	0.058	0.011	0.013	**0.164**	0.074	0.034	0.011	0.034	0.011	0.120
StMarks	0.152	0.137	0.126	0.040	0.061	0.034	0.017	**0.235**	0.157	0.030	0.033	0.032	0.034	0.184
Terrorist	**0.292**	0.244	0.276	0.056	0.157	0.055	0.010	0.106	**0.291**	0.008	0.058	0.031	0.055	**0.294**
Terrorist Train Bombing	0.680	0.572	0.670	0.426	0.191	0.497	0.063	0.224	**0.707**	0.014	0.493	0.036	0.497	**0.707**
US Air 97	0.392	0.372	0.391	0.098	0.038	0.073	0.007	0.321	**0.455**	0.004	0.053	0.011	0.073	0.450
Zakarays Karate Club	0.135	0.131	**0.192**	0.002	0.022	0.002	0.005	0.071	0.143	0.017	0.001	0.026	0.002	0.155
Average significant rank	3.788	5.600	4.612	9.725	8.338	9.425	12.150	5.688	2.975	11.650	9.300	10.475	9.300	**1.975**

Table 13. Results of statistical significance test of the difference in mean ranks against local methods. The results reported are those of the two-sample, one-tailed Mann-Whitney-Wilcoxon test of the mean ranks based on top-precision. For each entry, the upper triangle of the table reports the test p-values adjusted using the Benjamini, Hochberg, and Yekutieli method. Results significant at 95% confidence level are reported in boldface text. The lower triangular part reports, when significant at 95% confidence level, the comparison of the mean significant rank with $p = 0.05$ between the algorithm in the row and the one in the column.

	ADA	CNE	CH	HDI	HPI	JID	LHN	PAT	RAL	RND	SAI	SHP	SOI	ALG1
ADA		**0.00**	0.26	**0.00**	**0.00**	**0.00**	**0.00**	0.32	0.15	**0.00**	**0.00**	**0.00**	**0.00**	**0.00**
CNE	$>$		**0.02**	**0.00**	**0.00**	**0.00**	**0.00**	1.00	**0.00**	**0.00**	**0.00**	**0.00**	**0.00**	**0.00**
CH		$<$		**0.00**	**0.00**	**0.00**	**0.00**	0.93	**0.00**	**0.00**	**0.00**	**0.00**	**0.00**	**0.00**
HDI	$>$	$>$	$>$		0.09	1.00	**0.00**	**0.00**	**0.00**	**0.01**	1.00	1.00	1.00	**0.00**
HPI	$>$	$>$	$>$			0.10	**0.00**	**0.00**	**0.00**	**0.00**	0.26	**0.00**	0.16	**0.00**
JID	$>$	$>$	$>$				**0.00**	**0.00**	**0.00**	**0.00**	1.00	0.59	1.00	**0.00**
LHN	$>$	$>$	$>$	$>$	$>$	$>$		**0.00**	**0.00**	1.00	**0.00**	**0.01**	**0.00**	**0.00**
PAT				$<$	$<$	$<$	$<$		**0.04**	**0.00**	**0.00**	**0.00**	**0.00**	**0.00**
RAL		$<$	$<$	$<$	$<$	$<$	$<$	$<$		**0.00**	**0.00**	**0.00**	**0.00**	**0.00**
RND	$>$	$>$	$>$	$>$	$>$	$>$		$>$	$>$		**0.01**	**0.01**	**0.00**	**0.00**
SAI	$>$	$>$	$>$				$<$	$>$	$>$	$<$		0.45	1.00	**0.00**
SHP	$>$	$>$	$>$		$>$		$<$	$>$	$>$	$<$			0.36	**0.00**
SOI	$>$	$>$	$>$				$<$	$>$	$>$	$<$				**0.00**
ALG1	$<$	$<$	$<$	$<$	$<$	$<$	$<$	$<$	$<$	$<$	$<$	$<$	$<$	

Table 14. Comparison of the proposed edge weight method against the RA1 and RA2 rules. We report top-precision averaged over 100 test runs where 10% of the edges are removed at each run and used as test set. For every network, the results having the best significant rank with $p = 0.05$ are shown in bold. The last row shows the average significant rank over all networks (the lower the better). The columns n and m contain the number of nodes and edges in the network.

Network	n	m	RA1	RA2	RND	ALG1
ACM2009 Contacts	113	2,196	0.104	0.197	0.052	**0.267**
C.Elegans Metabolic	453	2,038	0.078	0.070	0.002	**0.187**
C.Elegans Neural	297	2,148	0.058	0.059	0.006	**0.107**
Centrality Literature	118	613	0.120	0.125	0.008	**0.174**
Chesapeake Lower	37	167	0.065	0.113	0.038	**0.194**
Chesapeake Middle	37	198	0.152	0.227	0.039	**0.264**
Chesapeake Upper	37	199	0.103	0.165	0.039	**0.298**
Codeminer	724	1,015	0.012	0.010	0.000	**0.054**
CPAN Authors	839	2,112	0.005	0.010	0.001	**0.106**
Cypress Dry	71	618	0.101	0.180	0.029	**0.196**
Cypress Wet	71	612	0.082	0.163	0.029	**0.216**
DNA Citation CC	35	59	0.002	0.005	0.010	**0.040**
DNA Citation	39	61	0.005	0.003	0.015	**0.040**
E.Coli	418	519	0.006	0.003	0.000	**0.036**
Erdos 971	433	1,314	0.086	0.097	0.002	**0.157**
Erdos 981	445	1,381	0.086	0.100	0.002	**0.151**
Erdos 991	454	1,417	0.089	0.100	0.001	**0.157**
Everglades	69	880	0.057	0.168	0.053	**0.236**
GD 01	259	640	0.091	0.097	0.002	**0.122**
Haggle Contact	274	2,124	0.371	0.149	0.007	**0.567**
Infectious	410	2,765	0.284	0.325	0.003	**0.342**
Japan Air	56	183	0.092	0.086	0.012	**0.228**
Jazz	198	2,742	0.337	0.368	0.016	**0.528**
Les Miserables	77	254	0.520	0.508	0.011	**0.554**
Macaque Neural	94	1,515	0.440	0.504	0.052	**0.556**
Manufacturing e-mail	167	3,250	0.145	0.310	0.030	**0.422**
Maspalomas	24	77	0.095	0.075	0.046	**0.169**
Narragan	35	204	0.109	0.154	0.046	**0.332**
Physicians	241	923	0.110	0.103	0.004	**0.124**
Polbooks	105	441	0.143	0.131	0.009	**0.213**
Political Blogs	643	2,280	0.036	0.070	0.001	**0.157**
Residence Hall	217	1,839	0.234	0.232	0.008	**0.246**
School	236	5,899	0.367	0.385	0.027	**0.407**
SFBD Food Web	128	2,106	0.089	0.074	0.034	**0.122**
SFBW Food Web	128	2,075	0.085	0.074	0.033	**0.119**
StMarks	54	350	0.073	0.114	0.029	**0.187**
Terrorist	62	152	0.162	0.197	0.007	**0.303**
Terrorist Train Bombing	64	243	0.581	0.569	0.009	**0.709**
US Air 97	332	2,126	0.109	0.212	0.005	**0.452**
Zakarays Karate Club	34	78	0.089	0.098	0.014	**0.142**
Average significant rank			2.725	2.362	3.913	**1.000**

Table 15. Results of statistical significance test of the difference in mean ranks against RA1 and RA2 rules. The results reported are those of the two-sample, one-tailed Mann-Whitney-Wilcoxon test of the mean ranks based on top-precision. For each entry, the upper triangle of the table reports the test p-values adjusted using the Benjamini, Hochberg, and Yekutieli method. Results significant at 95% confidence level are reported in boldface text. The lower triangular part reports, when significant at 95% confidence level, the comparison of the mean significant rank with $p = 0.05$ between the algorithm in the row and the one in the column.

	RA1	RA2	RND	ALG1
RA1		**0.00**	**0.00**	**0.00**
RA2	$<$		**0.00**	**0.00**
RND	$>$	$>$		**0.00**
ALG1	$<$	$<$	$<$	
